# Dictionary learning based noisy image super-resolution via distance penalty weight model

**DOI:** 10.1371/journal.pone.0182165

**Published:** 2017-07-31

**Authors:** Yulan Han, Yongping Zhao, Qisong Wang

**Affiliations:** Department of Automatic Test and Control, Harbin Institute of Technology, Harbin, Heilongjiang, China; Soochow University, CHINA

## Abstract

In this study, we address the problem of noisy image super-resolution. Noisy low resolution (LR) image is always obtained in applications, while most of the existing algorithms assume that the LR image is noise-free. As to this situation, we present an algorithm for noisy image super-resolution which can achieve simultaneously image super-resolution and denoising. And in the training stage of our method, LR example images are noise-free. For different input LR images, even if the noise variance varies, the dictionary pair does not need to be retrained. For the input LR image patch, the corresponding high resolution (HR) image patch is reconstructed through weighted average of similar HR example patches. To reduce computational cost, we use the atoms of learned sparse dictionary as the examples instead of original example patches. We proposed a distance penalty model for calculating the weight, which can complete a second selection on similar atoms at the same time. Moreover, LR example patches removed mean pixel value are also used to learn dictionary rather than just their gradient features. Based on this, we can reconstruct initial estimated HR image and denoised LR image. Combined with iterative back projection, the two reconstructed images are applied to obtain final estimated HR image. We validate our algorithm on natural images and compared with the previously reported algorithms. Experimental results show that our proposed method performs better noise robustness.

## 1 Introduction

Single image super-resolution (SR) is a classical problem in computer vision. In general, it uses signal processing techniques to recover a high resolution (HR) image from only one low resolution (LR) image. SR methods can be broadly classified into three categories: interpolation-based methods, reconstruction-based methods, and example-based methods.

Interpolation-based SR such as [1, 2] has been proposed for in various applications and it demonstrates the advantage of fast computational simplicity. But they usually fail to generate fine details in discontinuous regions and often result in introducing blurring of edges and other high-frequency features in practice [3].

Reconstruction-based methods usually integrate one or more sophisticated priors such as gradient profile prior [4], edge prior [5], and total variation [6] into SR literature to estimate the missed details. Recently, sparse-based regularization [7–10] has also been shown to be particularly effective for the ill-posed problems of SR. Usually, these methods achieved impressive results in preserving sharper edges and suppressing aliasing artifacts. However, the performance depends heavily upon a rational prior imposed on the up-sampled image [11].

Over the years, many example-based SR methods [12–14] have been proposed with demonstrated promising results and become the mainstream approaches of SR domain. The methods assume that the missing high frequency details can be estimated based on learning the mapping relationship from LR-HR patch pairs of external database and input LR patches. Two kinds of relationship models exist for these methods. One is that between LR patches and the corresponding HR patches in the database. After Freeman et al. [15] used Markov network to model the relationship, regression functions [16] are employed to exploit the relationship between HR and LR patch pairs. In addition, supervised or semi-supervised learning models are introduced into some of the algorithms [17–19]. Recently, a mapping of LR-HR image pairs was learned using a deep convolutional neural network [20], and has shown favorable results. D. Dai et al. [21] jointly learned a collection of regressors from LR to HR patches, which collectively yielded the smallest error for all training data. The other is that between LR example patches and input LR patches. Most of the methods [22, 23] is based on Nearest Neighbor Embedding (NNE). In these methods, a fixed number of nearest neighbors are extracted from database for each input LR patch, and then the corresponding HR patches are used to estimate the output HR patch by a linear combination determined by LR patch and its neighbors. Despite the algorithms are demonstrated by successful results, they highly depend on the number of neighbors which is difficult to determine. For this problem, [24] operates on a dynamic k-nearest neighbor algorithm, where k is small for test point with highly relevant neighbors and large others. Some researchers calculate the distance between input patch and its neighbors respectively. The neighbors will be abandoned when the distance is smaller than mean value. Yang [25] exploited sparse coding to perform image SR. The algorithm assumes that LR-HR patch pairs share the same sparse coefficients with respect to their respective dictionaries which are jointly learned from a set of external training images. It can be considered as neighbor embedding in sparse domain without choosing the number of neighbors. Since then, sparse coding is applied to SR problem [21–23], and achieves impressive results. Zeyde [26] used dimensionality reduction and orthogonal matching pursuit for sparse representation to improve efficiency. S. Wang [27], proposed a semi-coupled dictionary learning model, under which a pair of dictionaries and a mapping function describing the relationship between sparse coefficients of LR-HR patch pairs will be simultaneously learned. In [28], kernel ridge regression is employed to connect sparse coefficients of LR-HR patch pairs. Kaibing Zhang [29] determine the relationship between LR image patches and HR image patches by assuming that LR image patches and HR image patches are share the same sparse coefficients. R. Timofte et al. [30] proposed a fast image SR method called anchored neighbourhood regression (ANR) which learns sparse dictionaries and regressors anchored to dictionary atoms. This algorithm is faster, while making no compromise on quality. R. Timofte et al. [31] then produced an improved variant of ANR. The study in [31] enhanced these features and anchored regressors for ANR. Instead of learning the regressors on the dictionary, their method uses the full training material. It obtained improved quality, and became the fastest method indisputably. S. Gu [32] proposed a convolutional sparse coding based SR method to address consistency issue. In addition, researches show that image structures tend to repeat themselves within and across scales. [33–35] exploits the self-similarity of structures in nature image and extracts the database directly from the LR input image instead of the external database. Good reconstruction quality relies on much additional memory and running time to build counterparts across different scales in a recursive scheme. Therefore, its application is limited.

Although the algorithms can results in better performance, most of the SR algorithms including other learning-based methods assume that the input LR image is noise-free. Such assumption is not in accord with real applications. The algorithms are less robustness to noisy image SR. So another challenge is the super-resolution for noisy images. While compared with SR on clear LR input images, less attention has been paid to develop effective SR algorithms for noisy ones. J. Xie [36] first employs an adaptively regularized Shock filter to tackle the jagged noise, and then perform SR for depth image. The disadvantage of such scheme is that the artifacts can be created in denoising process and magnified in super-resolution process. Therefore, researchers started on simultaneously denoising and super-resolution. In [37], LR training images are magnified by a TV regularization model with a constraint before dictionaries training stage. However, the level of noise dealt with the method is small. Furthermore, it focuses on magnification only. Based on the current research status, we devote to design an algorithm to complete SR and denoising in the same framework to deal with noisy image patches.

**Fig 1:**
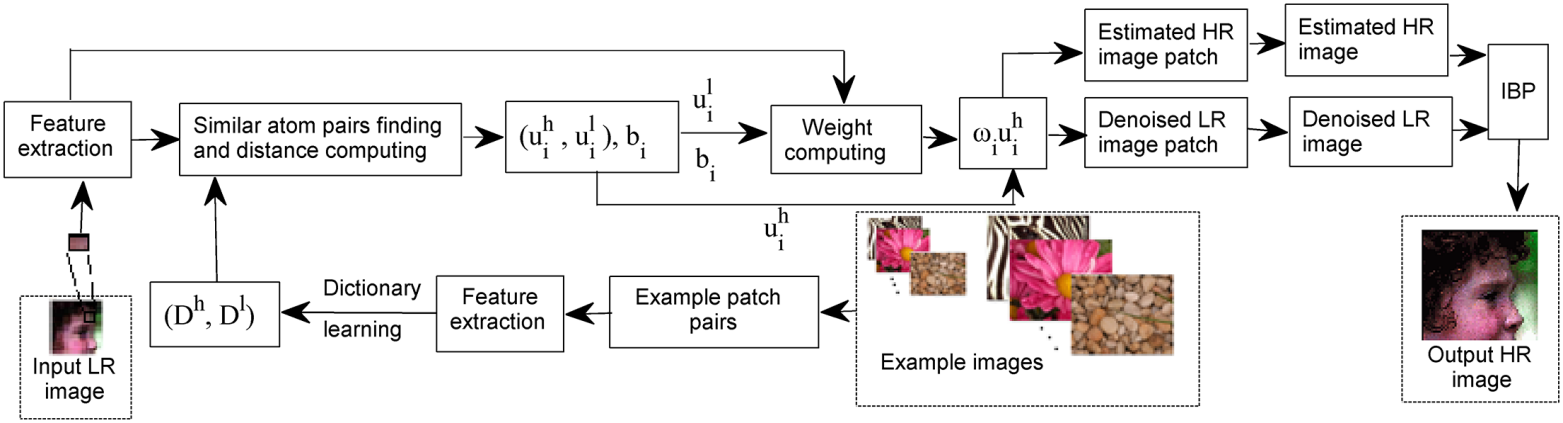
The flowchart of the proposed SR algorithm.

Sparse representation makes the signal energy only concentrated in a few atoms. Because of the special nature, some sparse coding based SR algorithms such as [25] show certain robustness to noisy image. In addition, sparse representation has been successfully employed in image denoising [38, 39], image restoration [40, 41] and other processing [42, 43]. The dictionary plays an important role in the sparse representation process. A predefined analytical dictionary (e.g., wavelet dictionary, Gabor dictionary) make the coding fast and explicit, but it is less effective to model the complex local structures of natural images. A synthesis dictionary (e.g., K-SVD dictionary) can be learned from example natural images and has more expensive computation but can better model complex image local structures [44]. In recent years, lots of dictionary learning methods have been proposed and achieved obvious performance. Feng et al. [45] propose to learn jointly the projection matrix for dimensionality reduction and the discriminative dictionary for face representation. Zhang et al. [46] propose a semisupervised label consistent dictionary learning framework for machine fault classification. Inspired by these, we introduce sparse theory to our research. The synthesis procedure is illustrated in Fig 1. The input LR image and example images are firstly cropped into patches. The example images are noise-free. Then the features of example patch pairs are extracted, which will be learned for dictionary pair. For each input LR patch, according to its features, it is easy to achieve simultaneously similar dictionary atom pairs $\left(u_{i}^{h},u_{i}^{l}\right)$ finding and calculating distance **b**_*i*_ between input LR patch and its similar atoms. Next, combined with the input LR image patch feature, LR dictionary atom $u_{i}^{l}$ and distance **b**_*i*_ are used to compute weight **ω**_*i*_. After the weight is computed, we can obtain estimated HR image patch and denoised LR image patch from $u_{i}^{h}\omega_{i}$. Put all the estimated HR patches into an estimated HR image, which is computed by averaging in overlapping regions. In the same way, we obtain the denoised LR image from all the denoised LR patches. At last, combined with the iterative back projection (IBP), the estimated HR image and the denoised LR image are applied to obtain the final output HR image.

The contributions can be summarized as follows.

(1) Different from the conventional methods, the proposed algorithm can process noisy image, and present for simultaneously image superresolution and denoising. Furthermore, in the training stage of our method, LR example images are noise-free. For different input LR images, even if the noise variance varies, the dictionary pair does not need to be retrained.

(2) The core idea of our proposed method is that the estimated HR patch is weighted average of similar HR example patches. To reduce computational cost for finding similar patches from millions of examples, example patches are replaced by the learned sparse dictionary which makes the signal energy only concentrate in few atoms.

(3) Penalty function is applied to least squares regression regularized by *l*_2_-norm for modeling weight. It makes the objective function treat each similar atom unequally. The function is determined by the similarity between input LR patch and its similar atom of LR dictionary. When the similarity is strong, we make the penalty small, which forces large weight at the same time. Conversely, when the similarity is weak, we make the penalty large, which forces small or zero weight at the same time.

(4) LR example patches subtracted mean pixel value are used for training dictionary rather than just their gradient features like other literatures such as [25]. In the training stage, for each LR example patch, we first subtract its mean pixel value, then connect it to its corresponding HR example patch into a single vector. All the new vectors are used as new HR examples to learn HR dictionary. Thus, the HR dictionary represents textures of HR example patches, but also that of LR example patches which are noise-free. Therefore, in the reconstruction stage, the HR dictionary can also be used to recover denoised input LR patches. This is different from conventional learning methods. Combined with iterative back projection (IBP), the denoised LR patches are applied to enhance robustness to noise.

The remainder of this paper is organized as follows. The proposed algorithm is presented in detail in Section 2. Experimental results and comparisons are demonstrated in section 3. Section 4 concludes this paper.

## 2 The proposed method

Firstly, let us recall the image degradation model which is shown in Eq (1). Given an observed LR image **Y** ∈ *R*^*M*^ that is a degraded version of a HR image **X** ∈ *R*^*N*^ of the same scene

$$\begin{array}{c} Y=G_{s}HX+v \end{array}$$ (1)

Where, **G**_*s*_ is the down-sampling operator with scaling factor *s*; **H** is the blurring operator; *v* is the noise. It is the task of SR reconstruction to recover **X** from **Y** as accurate as possible. It is considered that the image is noise-free by conventional SR methods.

### 2.1 Example database

From the example images $\left\{I_{1}^{h},I_{2}^{h},...,I_{N}^{h}\right\}$, LR images $\left\{I_{1}^{l},I_{2}^{l},...,I_{N}^{l}\right\}$ are first obtained, which are considered as noise-free ones. For each image $I_{j}^{h}$, its corresponding LR image $I_{j}^{l}$ is determined by

$$\begin{array}{c} I_{j}^{l}=G_{s}HI_{j}^{h} \end{array}$$ (2)

A set $\left\{p_{1}^{h},p_{2}^{h},...,p_{n}^{h}\right\}$ of vectorized HR patches of size $\sqrt{w}\times \sqrt{w}$ are taken from example HR images $\left\{I_{1}^{h},I_{2}^{h},...,I_{N}^{h}\right\}$ and a set $\left\{p_{1}^{l},p_{2}^{l},...,p_{n}^{l}\right\}$ of vectorized LR patches of size $\sqrt{w}/s\times \sqrt{w}/s$ are taken from example LR images $\left\{I_{1}^{l},I_{2}^{l},...,I_{N}^{l}\right\}$. Consequently, we obtain a database of HR-LR patch pairs

$$\begin{array}{c} \left(p^{h},p^{l}\right)=\left\{\left(p_{i}^{h},p_{i}^{l}\right)\in R^{w}\times R^{w/s^{2}},i=1,2,...,n\right\} \end{array}$$ (3)

### 2.2 Distance penalty weight model

For the super-resolution, given a LR image **Y**^*L*^, which is generated form HR image **X**^*H*^ by Eq (1), the task is to recover the unknown **X**^*H*^ from **Y**^*L*^ with the help of example patch pairs. The algorithm is performed with patch for the unit. Similar to [25], **Y**^*L*^ is firstly divided into overlapping patches

$$\begin{array}{c} Y^{L}=\left\{y_{i}^{l},i=1,2,...,N_{y}\right\} \end{array}$$ (4)

Where, $y_{i}^{l}$ is the vectorized LR image patch of size $\sqrt{w}/s\times \sqrt{w}/s$, *N*_*y*_ is the number of patches of **Y**^*L*^.

The estimated vectorized HR image **X**^*H*^ can be represented as

$$\begin{array}{c} X^{H}=\left\{x_{i}^{h},i=1,2,...,N_{y}\right\} \end{array}$$ (5)

Where,$x_{i}^{h}$ is the estimated HR image patch of size $\sqrt{w}\times \sqrt{w}$.

According to Eq (1), the relationship can be described by

$$\begin{array}{c} y_{i}^{l}=G_{s}Hx_{i}^{h}+v_{i} \end{array}$$ (6)

Where,**v**_*i*_ is the noise. We assume that it is Gaussian noise with zero-mean and variance *σ*^2^.

Thus, it become the purpose of super-resolution to estimate HR image patch $x_{i}^{h}$ from input LR image patch $y_{i}^{l}$.

As we known, for each $x_{i}^{h}$, it can be approximated by HR example patches through weighted average, which have similar structures. Therefore, based on this core idea, the problem in this method is to find the similar patches of $x_{i}^{h}$ in database and to calculate the weight.

Due to the repetition of local structures of images, a subset of patches $\left(u_{i}^{h},u_{i}^{l}\right)\in \left(p^{h},p^{l}\right)$ in which $u_{i}^{h}$ has similar structures with $x_{i}^{h}$ exists. That is

$$\begin{array}{c} x_{i}^{h}=\sum_{j=1}^{k}u_{ij}^{h}\omega_{ij}=u_{i}^{h}\omega_{i} \end{array}$$ (7)

Where, weight vector is **ω**_*i*_ = [*ω*_*i*1_, *ω*_*i*2_, …, *ω*_*ij*_, …, *ω*_*ik*_]^*T*^, *k* is the number of the patch pairs in this subset $\left(u_{i}^{h},u_{i}^{l}\right)$.

There are many methods to determine the weight, such as set the weights to be inversely proportional to the distance between patches. These methods relying on number of similar patches heavily, and cannot suppress noise. Now, we discuss a new weight model in details. According to the degradation model Eqs (1) and (7), we have

$$\begin{array}{c} G_{s}Hx_{i}^{h}=G_{s}Hu_{i}^{h}\omega_{i}=u_{i}^{l}\omega_{i} \end{array}$$ (8)

From Eq (8), we can obtain

$$\begin{array}{c} y_{i}^{l}=G_{s}Hx_{i}^{h}+v_{i}=u_{i}^{l}\omega_{i}+v_{i} \end{array}$$ (9)

Where, **v**_*i*_ is assumed as Gaussian noise with zero-mean and variance *σ*^2^.

Thus,

$$\begin{array}{c} y_{i}^{l}-u_{i}^{l}\omega_{i}=v_{i} \end{array}$$ (10)

$$\begin{array}{c} {∥y_{i}^{l}-u_{i}^{l}\omega_{i}∥}_{2}^{2}\leq \varepsilon_{i} \end{array}$$ (11)

Where, *ε*_*i*_ is related to *σ*^2^. We can see that the LR patch $y_{i}^{l}$ can be represented by the same weight vector **ω**_*i*_ over $u_{i}^{l}$, with an error *ε*_*i*_. That is to say, we can get the weight from input LR image patch and similar LR example patches with a controlled error.

Based on the above discussions, We formulate the weight solution as a least squares regression regularized by l2-norm:

$$\begin{array}{c} \omega_{i}=argmin{∥\omega_{i}∥}_{2}^{2}\;s.t.{∥y_{i}^{l}-u_{i}^{l}\omega_{i}∥}_{2}^{2}\leq \varepsilon_{i},\sum_{j=1}^{k}\omega_{ij}=1 \end{array}$$ (12)

From Eq (12), the objective function treats the patches $u_{i}^{l}$ equally. It is not flexible to obtain accurate weights for the input patch. Motivated by this, we introduce distance penalty to the least square problem

$$\begin{array}{c} \omega_{i}=argmin{∥b_{i}\cdot \omega_{i}∥}_{2}^{2}\;s.t.{∥y_{i}^{l}-u_{i}^{l}\omega_{i}∥}_{2}^{2}\leq \varepsilon_{i},\sum_{j=1}^{k}\omega_{ij}=1 \end{array}$$ (13)

Where, ⋅ denotes a point wise vector product, **b**_*i*_ = [*b*_*i*1_, *b*_*i*2_, …, *b*_*ij*_, …, *b*_*ik*_]^*T*^. **b**_*i*_ is the distance between $y_{i}^{l}$ and each similar example patch in $u_{i}^{l}$. When the similarity between $u_{ij}^{l}$ and $y_{i}^{l}$ is strong, we make the *b*_*ij*_ small, which forces large *ω*_*ij*_ at the same time. Conversely, when the similarity is weak, we make *b*_*ij*_ large, which forces small or zero *ω*_*ij*_ at the same time. It is simply determined by the squared Euclidean distance.

Eq (13) can be written as

$$\begin{array}{c} \omega_{i}=argmin{∥y_{i}^{l}-u_{i}^{l}\omega_{i}∥}_{2}^{2}+\lambda {∥b_{i}\cdot \omega_{i}∥}_{2}^{2}\;s.t.\sum_{j=1}^{k}\omega_{ij}=1 \end{array}$$ (14)

Where, *λ* is a regularization parameter.

According to Eq (10), we have

$$\begin{array}{c} {∥y_{i}^{l}-u_{i}^{l}\omega_{i}∥}_{2}^{2}\approx {∥v_{i}∥}_{2}^{2}\approx \gamma \sigma^{2} \end{array}$$ (15)

Where, *γ* is a positive constant. So we set *λ* = *γσ*^2^, when *σ* ≠ 0.

Thus, the main task in reconstruction stage is to find the patches $u_{i}^{l}$ from **p**^*l*^, which is similar to $y_{i}^{l}$ and compute the weight. Squared Euclidean distance can be adopted in to quantify the similarity. The corresponding $u_{i}^{h}$ is assumed to have similar structures with $x_{i}^{h}$. But it is uneasy to find similar patches for each input patch from millions of example patch pairs. It will take lots of time for the repetitive computation. Sparse dictionary make the signal energy only concentrate in few atoms, and some sparse coding based SR algorithm [25] show certain robustness to noisy image, so that we use a learned sparse dictionary instead of examples. We find similar patch pairs $\left(u_{i}^{h},u_{i}^{l}\right)$ from dictionary atom pairs, meaning $\left(u_{i}^{h},u_{i}^{l}\right)\in \left(D^{h},D^{l}\right)$.

Two dictionaries **D**^*h*^ and **D**^*l*^ are trained to have the same sparse coding for each HR and LR patch pair. Similar to Yang [25] and Chang [22], we subtract the mean pixel value for each HR example patch, so that the dictionary **D**^*h*^ represents image textures rather than absolute intensities. In the reconstruction stage, the mean value for each estimated patch is then predicted by its LR version. Also we employ first- and second-order derivatives as the feature extraction for LR example patches to train. Thus, *D*^*l*^ represents the gradient feature of images rather than absolute intensities. The four filters used here are:

$$\begin{array}{c} f_{1}=\left[-1,0,1\right],f_{2}=f_{1}^{T},f_{3}=\left[-1,0,2,0,1\right],f_{4}=f_{3}^{T} \end{array}$$ (16)

In addition, to enhance robustness to noise, we also subtract mean pixel value for each LR example patch, and connect the LR example patch to its corresponding HR example patch into a single vector, which is also used to learn **D**^*h*^. Thus, dictionary **D**^*h*^ represents textures of HR example patches, but also that of LR example patches which are noise-free. In the reconstruction stage, the **D**^*h*^ can also be used to recover denoised input LR patches. This is different from conventional learning methods.

From above, the training set is obtained by

$$\begin{array}{c} \left(P^{H},P^{L}\right)=\left\{\left(P_{i}^{H},P_{i}^{L}\right)=\left(\left[\begin{array}{c} p_{i}^{h}-{\bar{p}}_{i}^{h}\\ p_{i}^{l}-{\bar{p}}_{i}^{l} \end{array}\right],F\left(p_{i}^{l}\right)\right),i=1,2,...,n\right\} \end{array}$$ (17)

Where, (**p**^*h*^,**p**^*l*^) is original HR-LR patch pairs in Eq(3), ${\bar{p}}_{i}^{h}$ is the mean value of $p_{i}^{h}$, ${\bar{p}}_{i}^{l}$ is the mean value of $p_{i}^{l}$, *F*(⋅) is the operator to get four gradient vectors by Eq (16) and connect the four vectors into a single vector.

The set (**P**^*H*^,**P**^*L*^) is used to jointly train the dictionaries as

$$\begin{array}{c} \left(D^{h},D^{l}\right)=\min_{D^{h},D^{l},\alpha }\left\{\frac{1}{N}{∥P^{H}-D^{h}\alpha ∥}_{2}^{2}+\frac{1}{M}{∥P^{L}-D^{l}\alpha ∥}_{2}^{2}+\lambda_{0}{∥\alpha ∥}_{1}\right\} \end{array}$$ (18)

Where, *N* and *M* are the vector dimensions of **P**^*H*^ and **P**^*L*^, respectively.

To solve the problem easily, Eq (18) can be rewritten as

$$\begin{array}{c} \tilde{D}=\min_{\tilde{D},\alpha }\left\{{∥\tilde{P}-\tilde{D}\alpha ∥}_{2}^{2}+\lambda_{0}{∥\alpha ∥}_{1}\right\} \end{array}$$ (19)

Where, $\tilde{D}=\left[\begin{array}{c} \frac{1}{\sqrt{N}}D^{h}\\ \frac{1}{\sqrt{M}}D^{l} \end{array}\right]$, $\tilde{P}=\left[\begin{array}{c} \frac{1}{\sqrt{N}}P^{H}\\ \frac{1}{\sqrt{M}}P^{L} \end{array}\right]$.

The minimization of Eq (19) is a typical patch-based sparse problem. Many methods can be used to solve it. Yang [25] proposed the framework and acquired good results. However, it takes a large amount of time to solve this sparse model. Zeyde [26] improve the execution speed by dimensionality reduction on the patches through PCA and Orthogonal Matching Pursuit for the Sparse coding. For sparse dictionaries learning, we use the approach of Zeyde [26].

Gradient features(see Eq (16)) of LR example patches are used to learn LR dictionary. *D*^*l*^ represents the image gradient feature and $u_{i}^{l}\in D^{l}$. Therefore, the weight model is rewritten by

$$\begin{array}{c} {\hat{\omega }}_{i}=argmin{∥F\left(y_{i}^{l}\right)-u_{i}^{l}{\hat{\omega }}_{i}∥}_{2}^{2}+\lambda {∥b_{i}\cdot {\hat{\omega }}_{i}∥}_{2}^{2}\;s.t.\sum_{j=1}^{k}{\hat{\omega }}_{ij}=1 \end{array}$$ (20)

Where, ${\hat{\omega }}_{i}$ is the weight.

This problem Eq (20) is *l*_2_-norm constraint. We solve it for ${\hat{\omega }}_{i}$ by taking $\frac{\partial L}{\partial {\hat{\omega }}_{i}}=0$. The closed-form solution is

$$\begin{array}{c} {\hat{\omega }}_{i}={\left({\left(u_{i}^{l}\right)}^{T}u_{i}^{l}+\lambda B_{i}\right)}^{-1}{\left(u_{i}^{l}\right)}^{T}F\left(y_{i}^{l}\right) \end{array}$$ (21)

Where, $L={∥F\left(y_{i}^{l}\right)-u_{i}^{l}{\hat{\omega }}_{i}∥}_{2}^{2}+\lambda {∥b_{i}\cdot {\hat{\omega }}_{i}∥}_{2}^{2}$, **B**_*i*_ is a *k* × *k* diagonal matrix,

$$\begin{array}{c} B_{i}\left(j,j\right)=b_{ij}\left(j=1,2,...k\right) \end{array}$$ (22)

The final optimal weight is obtained by rescaling it so that $\sum_{j=1}^{k}{\hat{\omega }}_{ij}=1$.

### 2.3 Reconstruction

Based on the above discussions, for each input $y_{i}^{l}$, we start by extracting its gradient features and finding *k* similar atom pairs $\left(u_{i}^{h},u_{i}^{l}\right)$. Because the dictionary atoms are learned basis vectors, we find the similar atoms based on the correlation between the LR dictionary atoms and input LR patch rather than the Euclidean distance. Now, we describe how to compute the correlation.

$F(y_{i}^{l})$ can be represented by dictionary $D^{l}=\left[d_{1}^{l},d_{2}^{l},...,d_{j}^{l},...,d_{nd}^{l}\right]$ ($d_{j}^{l}$ is the LR dictionary atom, *nd* is dictionary size)

$$\begin{array}{c} F\left(y_{i}^{l}\right)=D^{l}\beta =\beta_{1}d_{1}^{l}+\beta_{2}d_{2}^{l}+...+\beta_{j}d_{j}^{l}+...+\beta_{nd}d_{nd}^{l} \end{array}$$ (23)

Where, **β** = [*β*_1_, *β*_2_, …, *β*_*j*_, …, *β*_*nd*_], *β*_*j*_ is the correlation between $d_{j}^{l}$ and $F(y_{i}^{l})$.

Eq (23) shows that every dictionary atom makes its own contribution to representing the input patch. The contribution of the *j*_*th*_ atom $d_{j}^{l}$ can be evaluated by *β*_*j*_. In other words, *β*_*j*_ is a measurement of the similarity between the input patch and the *j*_*th*_ dictionary atom. We consider that the larger the *β*_*j*_, the larger scale of similarity between input patch $F(y_{i}^{l})$ and dictionary atom $d_{j}^{l}$; and a small *β*_*j*_ means that there is little similarity. We can solve **β** by

$$\begin{array}{c} \beta ={\left(D^{l}\right)}^{T}F\left(y_{i}^{l}\right) \end{array}$$ (24)

Thus, ${\left(D^{l}\right)}^{T}F\left(y_{i}^{l}\right)$ could return the correlation. In Eq (20), we use distance **b**_*i*_ as the penalty. When the similarity between $F(y_{i}^{l})$ and $d_{j}^{l}$ is strong, we make the *b*_*ij*_ small, which forces large ${\hat{\omega }}_{ij}$ at the same time. Conversely, when the similarity is weak, we make *b*_*ij*_ large, which forces small or zero ${\hat{\omega }}_{ij}$ at the same time. Therefore, we use the reciprocal of *β*_*j*_ to compute the penalty. The atom pairs corresponding to the maximal *k* correlation coefficients constitute $\left(u_{i}^{h},u_{i}^{l}\right)$. **b**_*i*_ in Eq (20) is determined by

$$\begin{array}{c} b_{i}=1./Sort\left(abs\left({\left(D^{l}\right)}^{T}F\left(y_{i}^{l}\right)\right),k\right) \end{array}$$ (25)

Where, *Sort*(**a**, *num*) is a function returning *num* top biggest values of vector **a**, *abs*(.) is absolute value operation. The scheme can achieve simultaneously similar atoms finding and distance computing. If *σ* = 0, after finding similar atoms, we set **b**_*i*_ = **1**.

After this, we can easily obtain the weight ${\hat{\omega }}_{i}$ by Eq (20) and $u_{i}^{h}{\hat{\omega }}_{i}$. According to section 2.2, the reconstructed vector $u_{i}^{h}{\hat{\omega }}_{i}$ represents the estimated HR patch and the denoised LR patch correspondent to $y_{i}^{l}$. And the estimated patch and the denoised patch are subtracted mean pixel value. Based on this, we have

$$\begin{array}{c} \left[\begin{array}{c} {\hat{x}}_{i}^{h}\\ {\hat{y}}_{i}^{l} \end{array}\right]=u_{i}^{h}{\hat{\omega }}_{i}+\left[\begin{array}{c} E\left({\hat{x}}_{i}^{h}\right)C_{1}\\ E\left({\hat{y}}_{i}^{l}\right)C_{2} \end{array}\right] \end{array}$$ (26)

Where, ${\hat{x}}_{i}^{h}$ is the estimation of $x_{i}^{h}$, ${\hat{y}}_{i}^{l}$ is the denoised patch of $y_{i}^{l}$, $C_{1}\in R^{w_{1}}$ is an all-one column vector, $C_{2}\in R^{w_{2}}$ is an all-one column vector, *w*_1_ is the size of ${\hat{x}}_{i}^{h}$, *w*_2_ is the size of ${\hat{y}}_{i}^{l}$, E(⋅) is the mean evaluation operator.

Noise here is assumed as zero-means, so

$$\begin{array}{c} E\left(y_{i}^{l}\right)=E\left(D_{s}Hx_{i}^{h}+v_{i}\right)=E\left(D_{s}Hx_{i}^{h}\right)+E\left(v_{i}\right)\approx E\left(D_{s}Hx_{i}^{h}\right) \end{array}$$ (27)

We can see that the noise has little effect on image mean. The mean of ${\hat{y}}_{i}^{l}$ and ${\hat{x}}_{i}^{h}$ could be estimated by the mean of $y_{i}^{l}$. Eq (26) can be written by

$$\begin{array}{c} \left[\begin{array}{c} {\hat{x}}_{i}^{h}\\ {\hat{y}}_{i}^{l} \end{array}\right]=u_{i}^{h}{\hat{\omega }}_{i}+\left[\begin{array}{c} E\left(y_{i}^{l}\right)C_{1}\\ E\left(y_{i}^{l}\right)C_{2} \end{array}\right] \end{array}$$ (28)

Put all estimated patches ${\hat{x}}_{i}^{h}$ into a HR image ${\hat{X}}^{H}$, which is computed by averaging in overlapping regions. In the same way, we obtain a denoised image ${\hat{Y}}^{L}$ from ${\hat{y}}_{i}^{l}$. In order to strengthen the reconstruction constraint Eq (1), we compute the final estimated HR image X* by

$$\begin{array}{c} X^{*}={∥X^{*}-{\hat{X}}^{H}∥}_{2}^{2}\;s.t.D_{s}HX^{*}={\hat{Y}}^{L} \end{array}$$ (29)

The iterative back-projection (IBP) method [32] is used to solve this optimization problem

$$\begin{array}{c} X_{t+1}^{*}=X_{t}^{*}+\left(\left({\hat{Y}}^{L}-D_{s}HX_{t}^{*}\right){↑}_{s}\right)*p \end{array}$$ (30)

Where, $X_{t}^{*}$ is the estimate of the HR image at the *t*_*th*_ iteration, ↑_*s*_ denote up-scaling by factor *s*, *p* is a symmetric Gaussian filter.

The entire SR process is summarized as Algorithm 1.

**Algorithm 1**: The Proposed SR Algorithm

**Input:** the sparse dictionaries **D**^*h*^ and **D**^*l*^; input LR image **Y**; number of similar atoms *k*; a positive constant *γ*;

**output:** HR image X*;

1: **for** each patch $y_{i}^{l}$ of **Y** **do**

2:  Extract the gradient features for $y_{i}^{l}$ by Eq (16).

3:  Find *k* similar atom pairs $\left(u_{i}^{h},u_{i}^{l}\right)$ and compute **b**_*i*_ by Eq (25).

4:  Solve Eq (21) for ${\hat{\omega }}_{i}$.

5:  Generate estimated HR patch ${\hat{x}}_{i}^{h}$ and denoised patch ${\hat{y}}_{i}^{l}$ by Eq (28).

6: **end for**

7: Put the patches ${\hat{x}}_{i}^{h},\;i=1,2,...,N_{y}$ and ${\hat{y}}_{i}^{l},\;i=1,2,...,N_{y}$ into an image ${\hat{X}}^{H}$ and ${\hat{Y}}^{L}$, respectively.

8: Perform IBP Eq (30) to obtain a HR image X*.

## 3 Experiments

In this section, we will show the robustness of the proposed algorithm to noise and compare the state-of-the-art methods [20, 22, 25, 26, 31, 32]. In the training stage, we used 77 standard natural images as training set. For testing, we used Set5 [20, 31], Set14 [20, 31] and B100 [20, 31] to evaluate the performance of upscaling factors ×2, ×3 and ×4, respectively. Set5 and Set14 contain 5 and respectively 14 images for super-resolution evaluation. B100 contains 100 testing images of Berkeley Segmentation Dataset called BSDS300.

All LR images (training or test images) are generated from the original HR images. Firstly, the original HR images are directly blurred and down-sampled. The MATLAB function “imresize” is used here to complete the process. The function “imresize” involved a smooth filtering before down-sampling. Similar to [7], the noise is generated by MATLAB function “randn”, and *σ* times noise is added to the blurred and down-sampled test images. It should be noted that LR example images for training dictionary are noise-free. For color images used in experiments, SR algorithms are performed only on luminance channel, because humans are more sensitive to illuminant changes. Therefore, we first changes channels into YCbCr ones and then apply our method to the Y channel. We interpolate the color layers (Cb, Cr) using bicubic interpolation.

### 3.1 Parameters

In this section, we analyze the main parameters of our algorithm. The standard settings we use are Set5 [20, 31] database, dictionary size 1024, *γ* = 0.08 and *k* = 24 for upscaling factor ×2, *k* = 8 for upscaling factor ×3, ×4. Peak signal-to-noise ratio (PSNR) and reconstruction time were used as the objective criteria.

#### 3.1.1 Regularization parameter

**Fig 2:**
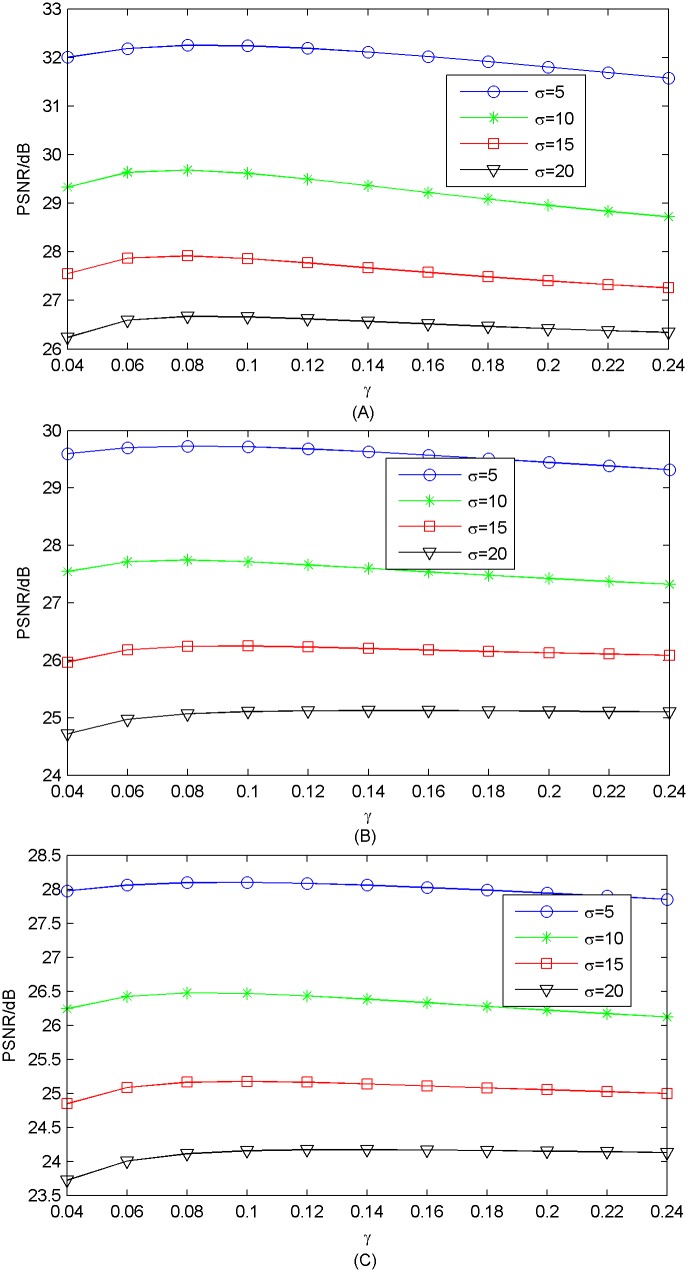
*γ* versus average PSNR on Set5. (A) upscaling factor ×2; (B) upscaling factor ×3; (C) upscaling factor ×4.

*γ* is a key regularization parameter of our method. Here, we validate the effectiveness of using different *γ*, and choose an appropriate one. The results of Set5 are shown in Fig 2. Experimental setting is dictionary size 1024 and *k* = 24 for upscaling factor ×2, *k* = 8 for upscaling factor ×3, ×4. We can see that the curves are not monotonic, and PSNR peaks at *γ* = 0.08. For different datasets, the optimal *γ* is slightly different (0.06 of Set14 and B100 compared to 0.08 of Set5) for reconstruction quality. The results of Set14 and B100 are shown in S1–S6 Figs. Therefore, we suggest determining *γ* to be around 0.08 in practice. Here, in all of our following experiments, we set *γ* as 0.08 for convenience.

#### 3.1.2 Dictionary size

**Fig 3:**
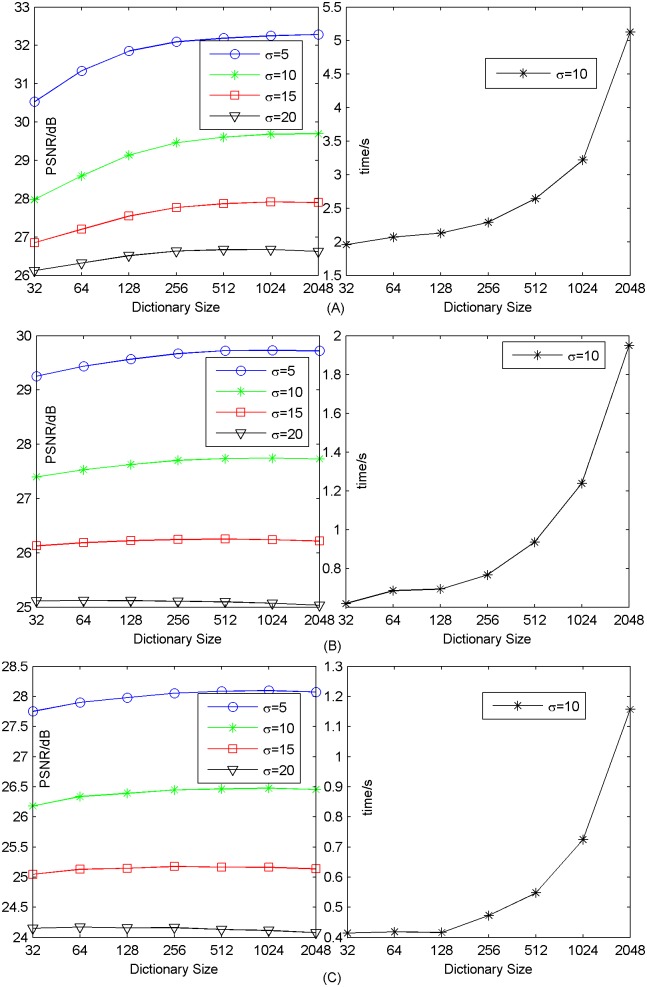
Dictionary size influence on performance on average on Set5. (A) upscaling factor ×2; (B) upscaling factor ×3; (C) upscaling factor ×4.

In this experiments, dictionary size is varied from 32 up to 2048, while the training samples are extracted from the same training images previously mentioned. In Fig 3, we present the results that show the relation between our method’s performance and the dictionary size when *γ* = 0.08 and *k* = 24 for upscaling factor ×2, *k* = 8 for upscaling factor ×3, ×4. Actually, noise has little effect on reconstruction time. So we only show the reconstruction time when *σ* = 10. We can see that the larger we learn the dictionary, the better reconstruction quality becomes. However, this comes with a higher computational cost. The result is the same as that of [25, 47]. Other datasets Set14 and B100 can also achieve similar results. The results of Set14 and B100 are shown in S7–S12 Figs. In practice, we suggest choosing the appropriate dictionary size as a tradeoff between reconstruction quality and computation. Dictionary size here is 1024 in our following experiments.

#### 3.1.3 Number of similar atoms

**Fig 4:**
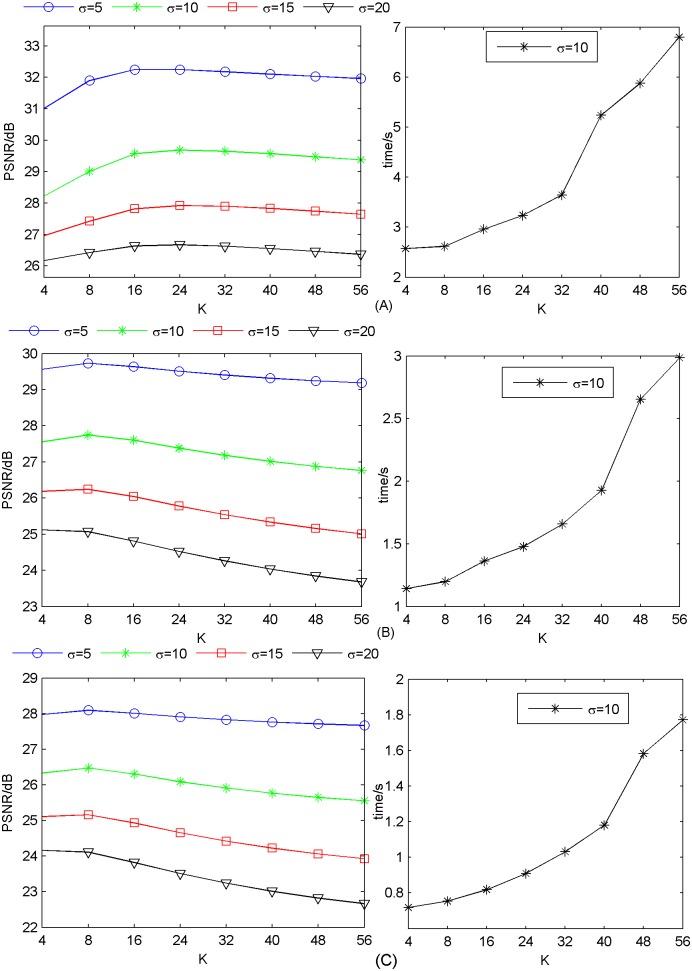
Number of similar atoms influence on performance on average on Set5. (A) upscaling factor ×2; (B) upscaling factor ×3; (C) upscaling factor ×4.

The proposed method finds the similar atom pairs for each input patch. The performance of the method depends on the number of similar atoms *k*. The effect of *k* is shown in Fig 4 when dictionary size is 1024 and *γ* = 0.08. Here, we also only show the reconstruction time when *σ* = 10. We can see that *k* = 24 is best for reconstruction quality when upscaling factor is ×2. The PSNR peaks at *k* = 8 when upscaling factor is ×3 or ×4. Moreover, average reconstruction time increases distinctly as *k* increases. It is due to the fact that by having a larger *k*, the computation of matrix inversion in Eq (21) increases. Other datasets Set14 and B100 can also achieve similar results. The results of Set14 and B100 are shown in S13–S18 Figs. Therefore, in resource-limited systems, a reasonable selection of *k* depends on the tradeoff between reconstruction quality and computational time. We will use *k* = 24 when upscaling factor is ×2, *k* = 8 when upscaling factor is ×3 or ×4 in our further experiments.

#### 3.1.4 Patch size and overlap

Intuitively, using a too large or too small patch size tends to produce a smooth or unwanted artifact as noticed also in [25, 29] and a larger overlapping leads to a better SR results [25]. Therefore, patch size is set as 6×6, 6×6 and 8×8 for upscaling factor ×2, ×3 and ×4, respectively, and overlap is set as 4, 3 and 4 for upscaling factor ×2, ×3 and ×4, respectively.

### 3.2 Performance evaluation

In this section we analyze the performance of our algorithm in quantitative and qualitative comparison with the state-of-the-art methods including NE [22], SCSR [25], Zeyde [26], A+ [31], SRCNN [20], and CSC [32]. We also show the reconstruction times of the algorithms. The code of the compared method was downloaded from the authors’ homepage. Peak signal-to-noise ratio (PSNR) and structural similarity (SSIM) were used as the objective criteria. The parameters are analyzed in the previous section. Besides the patch size and overlap(see section 3.1.4), the other parameter are unified (*γ* = 0.08, dictionary size = 1024, *k* = 24 for upscaling factor ×2, *k* = 8 for upscaling factor ×3 and ×4).

#### 3.2.1 Quality

**Fig 5:**
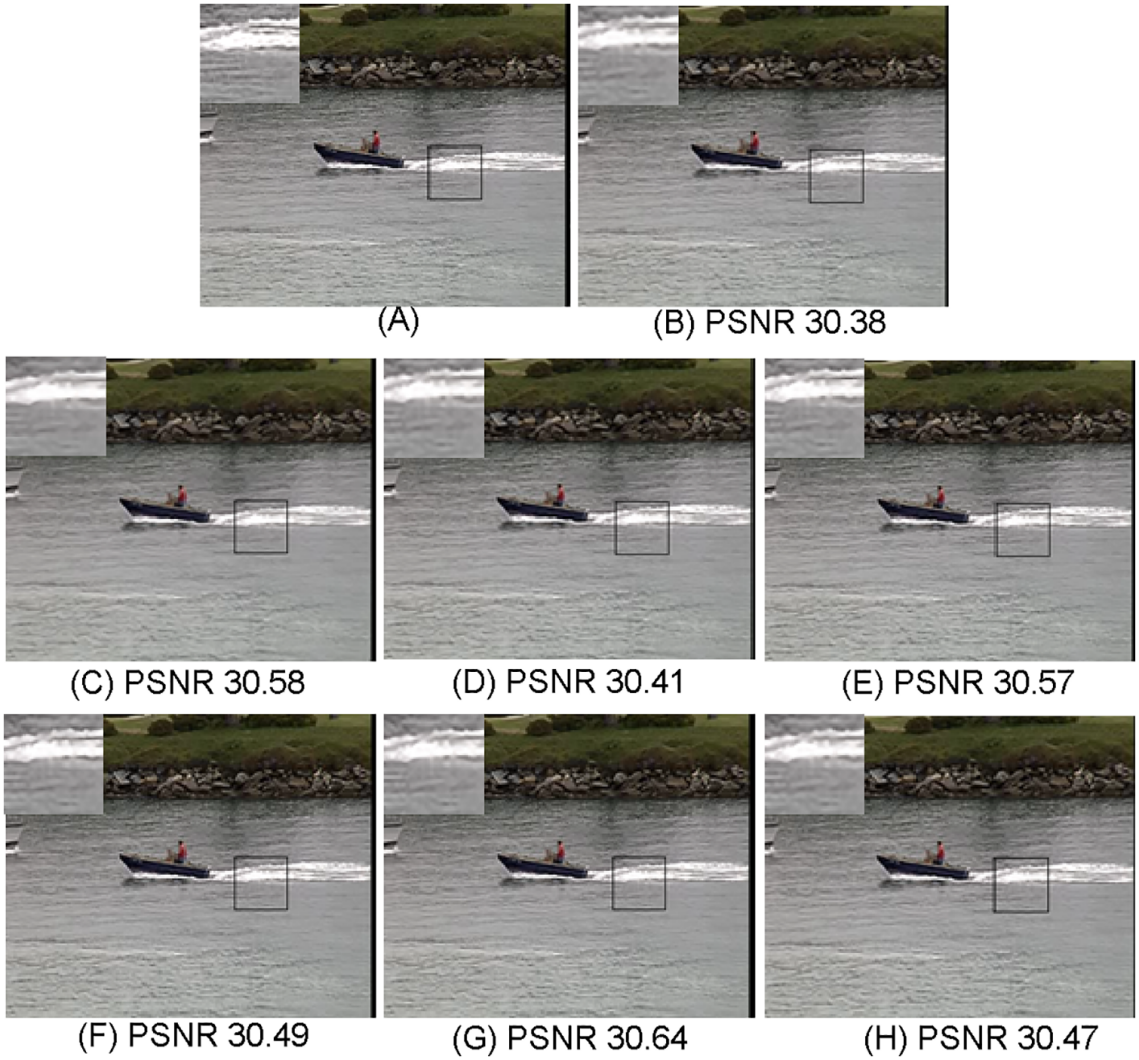
Comparisons with various image super-resolution methods on “coastguard” from Set14 with upscaling factor ×2 (*σ* = 0, PSNR in dB). (A) Ground truth HR; (B) NE [22]; (C) SCSR [25]; (D) Zedye [26]; (E) A+ [31]; (F) SRCNN [20]; (G) CSC [32]; (H) ours.

**Fig 6:**
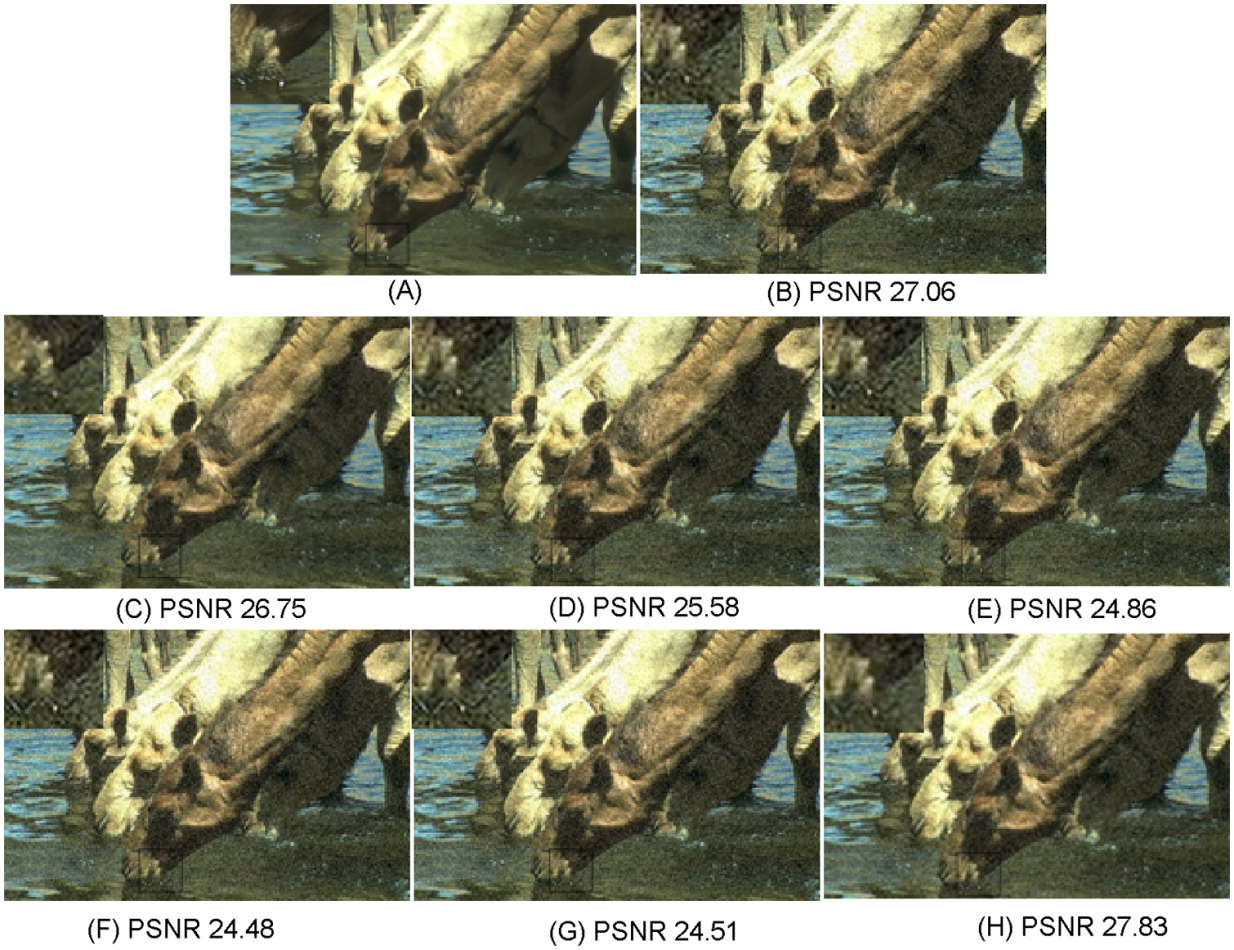
Comparisons with various image super-resolution methods on “16077” from B100 with upscaling factor ×2 (*σ* = 10, PSNR in dB). (A) Ground truth HR; (B) NE [22]; (C) SCSR [25]; (D) Zedye [26]; (E) A+ [31]; (F) SRCNN [20]; (G) CSC [32]; (H) ours.

**Fig 7:**
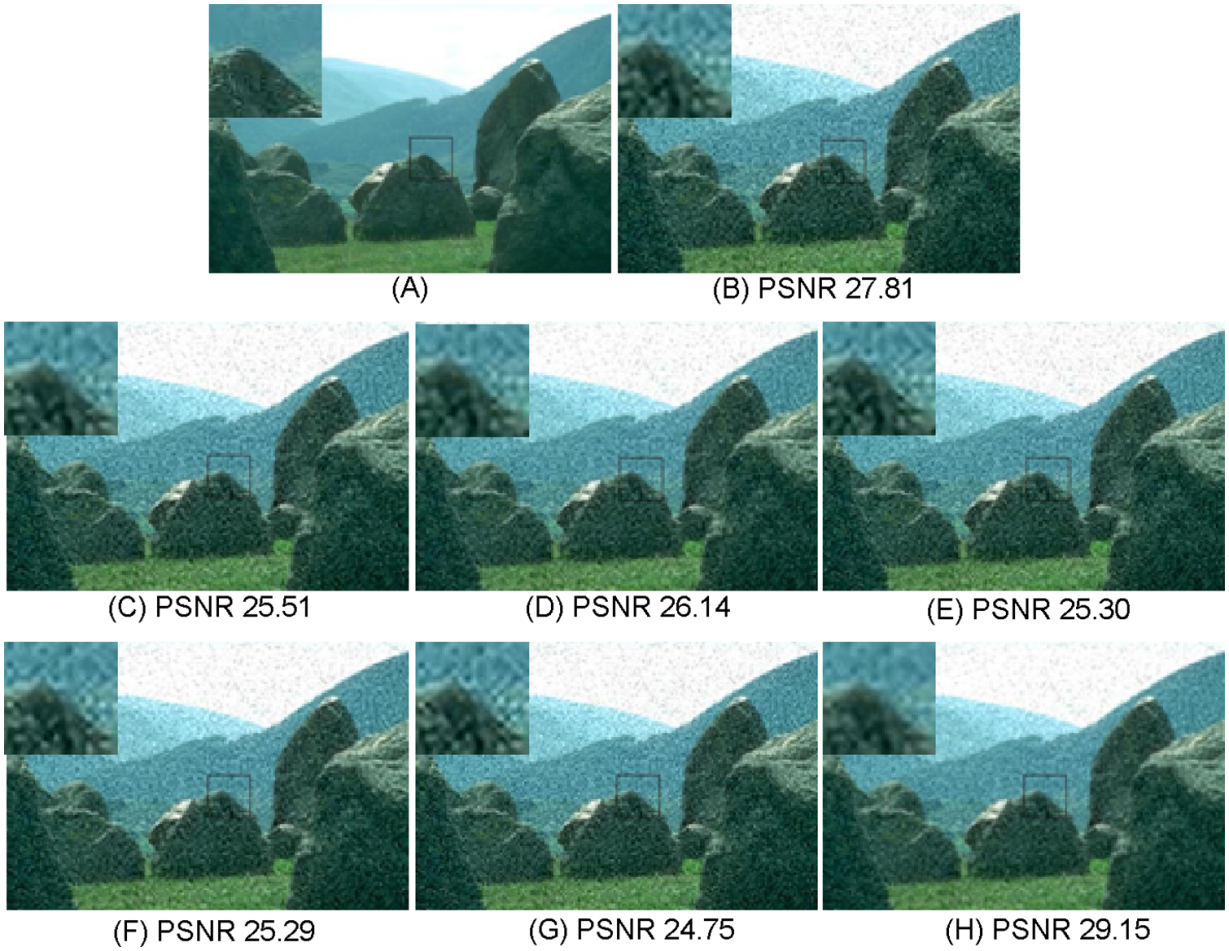
Comparisons with various image super-resolution methods on “241004” from B100 with upscaling factor ×3 (*σ* = 10, PSNR in dB). (A) Ground truth HR; (B) NE [22]; (C) SCSR [25]; (D) Zedye [26]; (E) A+ [31]; (F) SRCNN [20]; (G) CSC [32]; (H) ours.

**Fig 8:**
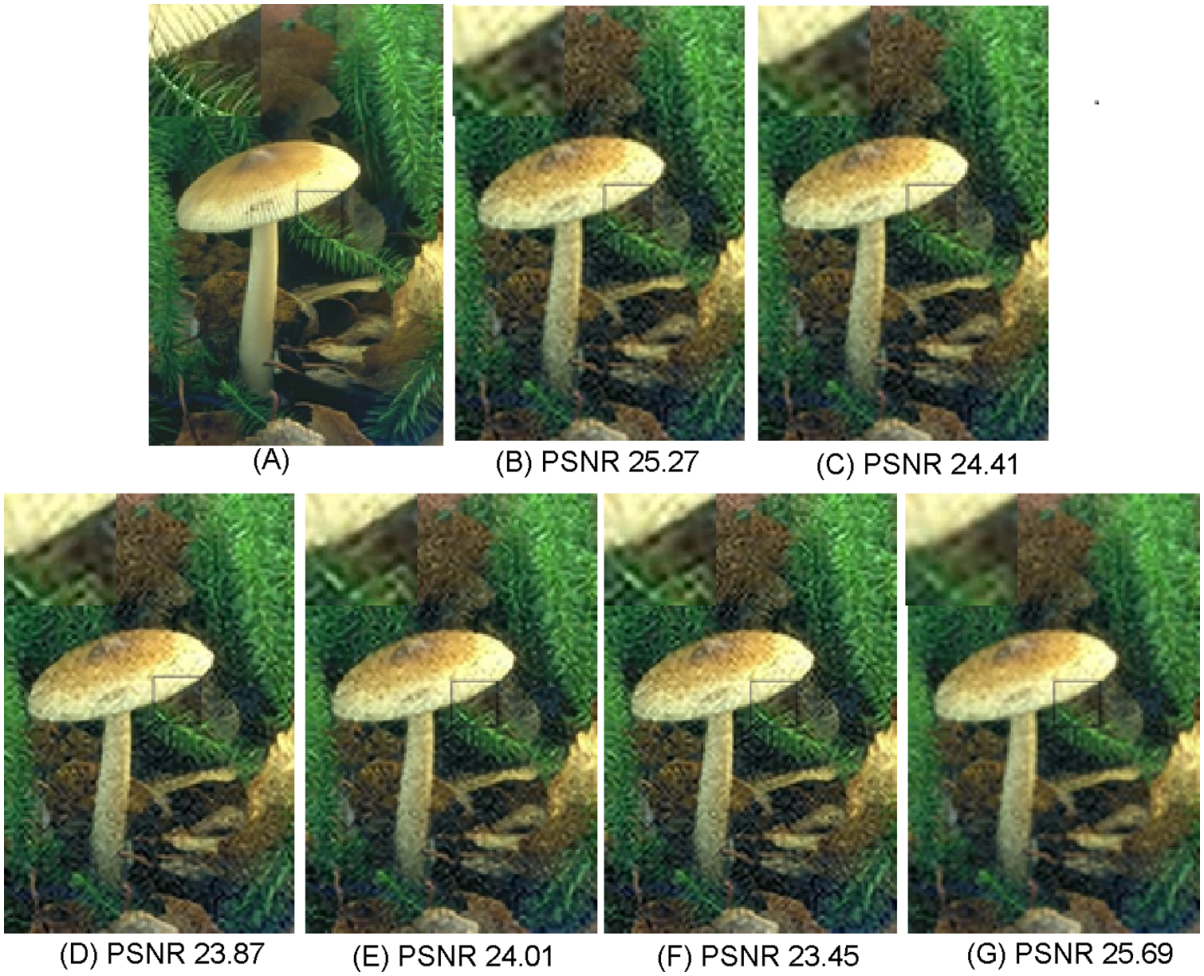
Comparisons with various image super-resolution methods on “208001” from B100 with upscaling factor ×4 (*σ* = 10, PSNR in dB). (A) Ground truth HR; (B) NE [22]; (C) Zedye [26]; (D) A+ [31]; (E) SRCNN [20]; (F) CSC [32]; (G) ours.

Tables 1–3 list the PSNR and SSIM comparisons. When *σ* = 0, the approach CSC [32] achieves the best performance. But it is not in accord with real application. When *σ* ≠ 0, as repeatedly shown, the results demonstrate the superiority of our proposed algorithm over other approaches on Set5, Set14 and B100. The average PSNR of the recent method CSC [32] is 0.24 dB (Set14, upscaling factor ×4, *σ* = 5) and 7.4 dB (Set5, upscaling factor ×2, *σ* = 20) behind our method. Compared with CSC, for dataset B100, the average PSNR improvement is from the minimum 0.52 dB (upscaling factor ×4, *σ* = 5) to the maximum 6.18 dB (upscaling factor ×2, *σ* = 20). In addition, our method improves on average 3.62 dB (Set5, upscaling factor ×2, *σ* = 20) over the next top robustness method SCSR [25]. Figs 5–8 provide a visual assessment. We can see that our method gets similar quality performance as the top methods it was compared to when *σ* = 0, and it has the strongest robustness.

**Table 1:** Comparisons of average PSNR (dB) and SSIM (*σ* = 0).

dataset	Scale	NE [22]		SCSR [25]		Zedye [26]		A+ [31]		SRCNN [20]		CSC [32]		ours	
		PSNR	SSIM	PSNR	SSIM	PSNR	SSIM	PSNR	SSIM	PSNR	SSIM	PSNR	SSIM	PSNR	SSIM
Set5	×2	35.77	0.949	36.04	0.951	35.78	0.949	36.55	0.954	36.34	0.952	**36.62**	**0.955**	35.65	0.948
	×3	31.84	0.896	31.40	0.887	31.90	9.897	32.59	0.909	32.39	0.887	**32.66**	**0.909**	31.57	0.895
	×4	29.61	0.840	-	-	29.69	0.843	30.28	0.860	30.09	0.853	**30.36**	**0.859**	29.49	0.841
Set14	×2	31.76	0.899	31.71	0.903	31.81	0.899	32.28	0.906	32.18	0.904	**32.31**	**0.907**	31.71	0.901
	×3	28.60	0.808	28.07	0.803	28.67	0.808	29.13	0.819	29.00	0.815	**29.15**	**0.821**	28.26	0.811
	×4	26.81	0.733	-	-	26.88	0.734	**27.32**	0.749	26.61	0.725	27.30	**0.750**	26.55	0.738
B100	×2	30.41	0.871	31.04	0.884	30.40	0.868	30.77	0.877	31.14	0.885	**31.27**	**0.888**	30.76	0.881
	×3	27.85	0.771	27.81	0.772	27.87	0.770	28.18	0.780	28.21	0.780	**28.31**	**0.786**	27.85	0.778
	×4	26.47	0.697	-	-	26.55	0.697	26.77	0.709	26.71	0.702	**26.83**	**0.711**	26.51	0.703

**Table 2:** The results of PSNR (dB) and SSIM on the set5 dataset.

Scale	*σ*	Set5 images	NE [22]		SCSR [25]		Zedye [26]		A+ [31]		SRCNN [20]		CSC [32]		ours	
			PSNR	SSIM	PSNR	SSIM	PSNR	SSIM	PSNR	SSIM	PSNR	SSIM	PSNR	SSIM	PSNR	SSIM
×2	5	baby	31.5	0.7556	32.96	0.8287	31.9	0.7753	31.11	0.7399	33.47	0.8482	30.63	0.7190	**34.19**	**0.8760**
		bird	31.84	0.8063	32.21	0.8683	32.25	0.8226	31.48	0.7918	33.34	0.8836	30.89	0.7704	**34.46**	**0.9134**
		butterfly	28.35	0.8372	**29.05**	0.8819	28.70	0.8492	28.97	0.8385	26.87	0.8588	28.43	0.8222	28.08	**0.8936**
		head	30.71	0.7022	32.04	0.763	31.08	0.7193	30.42	0.6898	32.34	0.774	29.99	0.6693	**32.87**	**0.7915**
		woman	30.39	0.7867	31.33	0.8465	30.68	0.803	30.3	1.7762	30.6	0.8575	29.82	0.7575	**31.65**	**0.8913**
		**average**	30.56	0.7776	31.52	0.8377	30.92	0.7939	30.46	0.9672	31.32	0.8444	29.95	0.7477	**32.25**	**0.8732**
	10	baby	26.17	0.5044	28.54	0.6364	26.66	0.5307	25.73	0.4834	25.43	0.4713	25.21	0.4587	**31.41**	**0.7946**
		bird	26.30	0.5702	28.51	0.6943	26.77	0.5946	25.84	0.5487	25.52	0.5373	25.24	0.5208	**30.99**	**0.8345**
		butterfly	25.04	0.6845	**26.33**	0.7572	25.41	0.7002	24.95	0.6777	24.52	0.6597	24.3	0.6547	26.08	**0.8381**
		head	25.92	0.4649	28.34	0.5936	26.4	0.4899	25.53	0.4463	25.44	0.4454	24.99	0.4192	**30.84**	**0.7124**
		woman	25.83	0.5698	27.68	0.6765	26.23	0.5912	25.44	0.5528	25.14	0.5433	24.92	0.5311	**29.11**	**0.8199**
		**average**	25.85	0.5588	27.88	0.6716	26.29	0.5813	25.50	0.5418	25.21	0.5314	24.93	0.517	**29.69**	**0.7999**
	15	baby	22.85	0.3496	25.62	0.4863	23.35	0.3726	22.39	0.3303	21.91	0.3108	21.83	0.3081	**29.71**	**0.7343**
		bird	22.93	0.4126	25.52	0.5495	23.41	0.4358	22.45	0.3915	22.03	0.3753	21.81	0.3643	**28.98**	**0.7723**
		butterfly	22.29	0.5741	23.88	0.6497	22.64	0.5892	21.92	0.5626	21.37	0.5399	21.3	0.5392	**24.23**	**0.7802**
		head	22.81	0.3150	25.64	0.4546	23.3	0.3372	22.4	0.2983	22.18	0.2933	21.7	0.2709	**29.50**	**0.6567**
		woman	22.69	0.4311	24.98	0.5442	23.12	0.4505	22.24	0.4137	21.81	0.3971	21.67	0.3933	**27.18**	**0.7592**
		**average**	22.71	0.4165	25.13	0.5369	23.164	0.4371	22.28	0.3993	21.86	0.3833	21.66	0.3752	**27.92**	**0.7406**
	20	baby	20.5	0.2556	23.46	0.3792	20.98	0.2741	20.03	0.2389	19.45	0.2196	19.39	0.2191	**28.51**	**0.6856**
		bird	20.55	0.3105	23.34	0.4398	21.02	0.3304	20.05	0.2914	19.57	0.2754	19.35	0.2665	**27.67**	**0.7219**
		butterfly	20.12	0.4935	21.95	0.5666	20.46	0.5071	19.64	0.4785	19.04	0.4555	19.03	0.4557	**22.87**	**0.7262**
		head	20.58	0.2253	23.58	0.3529	21.05	0.2429	20.15	0.211	19.82	0.2049	19.29	0.185	**28.47**	**0.6121**
		woman	20.41	0.3415	22.93	0.4475	20.83	0.3576	19.94	0.3247	19.42	0.3062	19.28	0.3051	**25.86**	**0.7077**
		**average**	20.43	0.3253	23.05	0.437	20.87	0.3424	19.96	0.3089	19.46	0.2923	19.27	0.2863	**26.67**	**0.6907**
×3	5	baby	30.70	0.7523	30.42	0.7483	31.06	0.7713	30.33	0.7349	30.5	0.7495	29.95	0.7142	**32.05**	**0.8407**
		bird	30.53	0.8043	30.22	0.8024	30.86	0.8214	30.39	0.793	30.45	0.8084	29.96	0.7724	**31.43**	**0.8756**
		butterfly	24.97	0.7859	24.8	0.7846	25.20	0.8006	**25.94**	0.8088	26.15	0.8044	25.53	0.7841	24.97	**0.8280**
		head	30.08	0.6777	30.01	0.6748	30.4	0.6938	29.78	0.6638	30.09	0.6871	29.42	0.6436	**31.40**	**0.7442**
		woman	28.33	0.7781	28.08	0.776	28.61	0.7943	28.6	0.7719	28.51	0.7823	28.2	0.7483	**28.80**	**0.8534**
		**average**	28.92	0.7597	28.71	0.7572	29.23	0.7763	29.01	0.7545	29.14	0.7663	28.61	0.7325	**29.73**	**0.8284**
	10	baby	26.08	0.5299	25.85	0.5257	26.54	0.5576	25.53	0.5034	25.59	0.5091	25.05	0.4762	**29.78**	**0.7625**
		bird	26.08	0.5952	25.83	0.5913	26.51	0.6215	25.55	0.5685	25.58	0.5798	25.03	0.5394	**28.99**	**0.7993**
		butterfly	23.22	0.6601	23.06	0.6602	23.5	0.6784	23.46	0.6665	23.45	0.6573	22.92	0.6341	**23.42**	**0.7635**
		head	25.89	0.4726	25.81	0.468	26.36	0.498	25.39	0.4488	25.61	0.4677	24.87	0.4199	**29.64**	**0.6749**
		woman	25.17	0.5863	24.94	0.5839	25.54	0.6100	24.86	0.5673	24.80	0.5734	24.34	0.5371	**26.89**	**0.7812**
		**average**	25.29	0.5688	25.10	0.5658	25.69	0.5931	24.96	0.5509	25.01	0.5575	24.44	0.5213	**27.74**	**0.7563**
	15	baby	22.95	0.3812	22.72	0.3763	23.41	0.406	22.34	0.3541	22.25	0.3506	21.79	0.3285	**28.13**	**0.6977**
		bird	23.00	0.4435	22.75	0.4375	23.44	0.4683	22.35	0.4125	22.23	0.4114	21.78	0.3835	**27.33**	**0.7353**
		butterfly	21.34	0.5608	21.11	0.5585	21.62	0.5781	21.12	0.556	20.99	0.5446	20.57	0.5246	**22.10**	**0.6994**
		head	22.98	0.3324	22.86	0.3273	23.44	0.3556	22.39	0.3083	22.34	0.3114	21.71	0.2778	**28.22**	**0.6178**
		woman	22.53	0.4519	22.28	0.4478	22.91	0.4733	21.99	0.4278	21.84	0.4257	21.43	0.4002	**25.43**	**0.7168**
		**average**	22.56	0.434	22.34	0.4295	22.96	0.4563	22.04	0.4117	21.93	0.4087	21.46	0.3829	**26.24**	**0.6934**
	20	baby	20.68	0.2856	20.43	0.2805	21.11	0.3056	20.04	0.2606	19.83	0.2528	19.4	0.2375	**26.78**	**0.6397**
		bird	20.73	0.3403	20.47	0.3337	21.16	0.4998	20.04	0.3109	19.8	0.3023	19.39	0.2825	**26.07**	**0.6782**
		butterfly	19.63	0.4847	19.36	0.4802	19.9	0.262	19.15	0.4708	18.98	0.4612	18.62	0.4435	**21.18**	**0.6453**
		head	20.83	0.243	20.69	0.238	21.27	0.3791	20.21	0.2215	19.96	0.2154	19.35	0.1913	**26.97**	**0.5650**
		woman	20.47	0.3614	20.19	0.3558	20.82	0.3615	19.82	0.3361	19.58	0.3286	19.19	0.3108	**24.35**	**0.6598**
		**average**	20.47	0.3430	20.23	0.3376	20.85	0.3616	19.85	0.3200	19.63	0.3121	19.19	0.2931	**25.07**	**0.6376**
×4	5	baby	29.79	0.7405	-	-	30.1	0.7579	29.57	0.7276	29.95	0.7566	29.18	0.7045	**30.65**	**0.8066**
		bird	29.19	0.7878	-	-	29.43	0.8029	29.22	0.7823	29.38	0.8053	28.86	0.7608	**29.67**	**0.8355**
		butterfly	22.92	0.7246	-	-	23.13	0.7414	**23.70**	**0.7624**	24.22	0.7734	23.46	0.7344	23.05	0.7582
		head	29.4	0.6561	-	-	29.69	0.6717	29.27	0.6494	29.67	0.6788	28.93	0.6309	**30.39**	**0.7093**
		woman	26.52	0.7496	-	-	26.76	0.7654	26.96	0.7522	26.83	0.7688	26.71	0.7282	**26.82**	**0.8062**
		**average**	27.56	0.7317	-	-	27.82	0.7479	27.74	0.7348	28.01	0.7566	27.43	0.7118	**28.10**	**0.7832**
	10	baby	25.73	0.5442	-	-	26.14	0.5697	25.25	0.5196	25.67	0.5523	24.72	0.4864	**28.66**	**0.7342**
		bird	25.61	0.6081	-	-	25.93	0.6310	25.16	0.5832	25.51	0.6208	24.61	0.5504	**27.56**	**0.7612**
		butterfly	21.78	0.6258	-	-	22.00	0.6427	22.02	0.6405	**22.41**	0.6546	21.62	0.6038	21.96	**0.7053**
		head	25.63	0.4798	-	-	26.04	0.5038	25.24	0.4619	25.77	0.5052	24.7	0.431	**28.82**	**0.6504**
		woman	24.21	0.5843	-	-	24.49	0.6056	24.05	0.5707	24.15	0.599	23.59	0.5376	**25.39**	**0.7394**
		**average**	24.59	0.5684	-	-	24.92	0.5906	24.34	0.5552	24.70	0.5864	23.85	0.5218	**26.48**	**0.7181**
	15	baby	22.79	0.4029	-	-	23.19	0.4264	22.21	0.3755	22.37	0.3935	21.59	0.3433	**27.19**	**0.6783**
		bird	22.82	0.4648	-	-	23.17	0.488	22.23	0.434	22.40	0.4625	21.58	0.3985	**26.11**	**0.7021**
		butterfly	20.38	0.5401	-	-	20.57	0.5544	20.20	0.5375	20.43	0.549	19.73	0.5025	**20.83**	**0.6482**
		head	22.85	0.3508	-	-	23.25	0.3722	22.36	0.3301	22.60	0.3628	21.63	0.2942	**27.53**	**0.6037**
		woman	21.98	0.4594	-	-	22.28	0.4788	21.53	0.438	21.57	0.4564	20.98	0.4057	**24.16**	**0.6822**
		**average**	22.16	0.4436	-	-	22.49	0.4640	21.71	0.4230	21.87	0.4448	21.10	0.3888	**25.16**	**0.6629**
	20	baby	20.59	0.3081	-	-	20.96	0.3271	19.97	0.2812	19.91	0.2885	19.25	0.2515	**25.98**	**0.6304**
		bird	20.68	0.3633	-	-	21.01	0.3819	20.03	0.3308	20.01	0.3485	19.28	0.2957	**25.01**	**0.6514**
		butterfly	18.99	0.4705	-	-	19.16	0.4819	18.54	0.4563	18.65	0.4652	18.03	0.4251	**19.97**	**0.5984**
		head	20.77	0.264	-	-	21.15	0.2819	20.21	0.2444	20.19	0.2643	19.31	0.2065	**26.38**	**0.5609**
		woman	20.1	0.3704	-	-	20.41	0.3865	19.53	0.3461	19.45	0.3570	18.87	0.3154	**23.22**	**0.6335**
		**average**	20.23	0.3553	-	-	20.54	0.3719	19.66	0.3318	19.64	0.3446	18.95	0.2988	**24.11**	**0.6149**

**Table 3:** The results of average PSNR (dB) and SSIM on the Set14 and B100 dataset.

dataset	Scale	*σ*	NE [22]		SCSR [25]		Zedye [26]		A+ [31]		SRCNN [20]		CSC [32]		ours	
			PSNR	SSIM	PSNR	SSIM	PSNR	SSIM	PSNR	SSIM	PSNR	SSIM	PSNR	SSIM	PSNR	SSIM
Set14	×2	5	28.74	0.7514	29.31	0.7981	29.01	0.7647	28.71	0.7737	28.61	0.7435	28.36	0.7275	**29.69**	**0.8205**
		10	25.08	0.5551	26.59	0.6478	25.46	0.5750	24.78	0.5400	24.45	0.5264	24.31	0.5180	**27.80**	**0.7381**
		15	22.29	0.4204	24.31	0.5213	22.71	0.4394	21.89	0.4309	21.42	0.3848	21.35	0.3821	**26.38**	**0.6732**
		20	20.15	0.3302	22.47	0.4259	20.57	0.3466	19.70	0.3140	19.14	0.2945	19.10	0.2933	**25.35**	**0.6220**
	×3	5	26.86	0.6903	26.55	0.6891	27.08	0.7035	26.96	0.6859	26.99	0.6942	26.70	0.6703	**27.16**	**0.7220**
		10	24.19	0.5240	23.95	0.5215	24.52	0.5441	23.92	0.5079	23.90	0.5014	23.53	0.4856	**25.78**	**0.6663**
		15	21.89	0.4032	21.64	0.3990	22.24	0.4221	21.43	0.3827	21.29	0.3781	20.96	0.3606	**24.67**	**0.6075**
		20	19.99	0.3196	19.72	0.3142	20.35	0.3355	19.43	0.2981	19.20	0.2900	18.88	0.2767	**23.77**	**0.5579**
	×4	5	25.57	0.6398	-	-	25.76	0.6526	25.76	0.6416	25.89	0.6575	25.49	0.6241	**25.73**	**0.6788**
		10	23.42	0.4985	-	-	23.42	0.5174	23.24	0.4865	23.45	0.5078	22.84	0.4607	**24.64**	**0.6171**
		15	21.39	0.3896	-	-	21.69	0.4076	21.01	0.3713	21.08	0.3836	20.51	0.3448	**23.70**	**0.5686**
		20	19.66	0.3115	-	-	19.96	0.3265	19.15	0.2910	19.08	0.2958	18.57	0.2655	**22.91**	**0.5283**
B100	×2	5	28.00	0.7264	28.81	0.7719	28.19	0.7380	27.96	0.7196	28.02	0.721	27.83	0.7076	**28.95**	**0.7917**
		10	24.66	0.5279	26.28	0.6200	25.02	0.5480	24.36	0.5123	24.17	0.5059	24.07	0.4997	**27.29**	**0.7037**
		15	22.01	0.3951	24.10	0.4941	22.42	0.4136	21.63	0.3792	21.25	0.3661	21.23	0.3654	**26.08**	**0.6378**
		20	19.95	0.3077	22.32	0.4000	20.37	0.3233	19.52	0.2925	19.03	0.277	19.02	0.2779	**25.20**	**0.5873**
	×3	5	26.32	0.6518	26.79	0.6728	26.49	0.6638	26.34	0.6463	26.46	0.6586	26.20	0.6351	**26.85**	**0.7010**
		10	23.86	0.4878	23.74	0.4874	24.15	0.5067	23.58	0.4716	23.60	0.4767	23.27	0.4538	**25.64**	**0.6273**
		15	21.66	0.3703	21.47	0.3674	21.99	0.3882	21.22	0.3510	21.10	0.3481	20.81	0.3326	**24.66**	**0.5702**
		20	19.82	0.2902	19.59	0.2855	20.17	0.3051	19.29	0.2706	19.07	0.2635	18.78	0.2526	**23.84**	**0.5223**
	×4	5	25.3	0.6015	-	-	25.46	0.6133	25.36	0.5991	25.53	0.6171	25.2	0.5857	**25.72**	**0.6414**
		10	23.23	0.4615	-	-	23.49	0.4800	23.01	0.4478	23.23	0.4690	22.69	0.4269	**24.74**	**0.5815**
		15	21.26	0.3562	-	-	21.56	0.3736	20.86	0.3378	20.95	0.3500	20.43	0.3161	**23.89**	**0.5358**
		20	19.56	0.2820	-	-	19.86	0.2966	19.06	0.2622	18.99	0.2669	18.52	0.2412	**23.15**	**0.4980**

#### 3.2.2 Reconstruction time

Average reconstruction time of test images in Set5 was compared when *σ* = 10. Actually, noise has little effect on test results. The experiments were conducted on the same computer. The results are summarized in Table 4. The reconstruction time varies a lot for different upscaling factors. Our algorithm cost fewer than 10s. The reconstruction time of our algorithm is comparable to that of SCSR, CSC, and SRCNN. SCSR is the slowest method.

**Table 4:** Comparisons of average reconstruction time (s)on Set5.

Scale	NE [22]	SCSR [25]	Zedye [26]	A+ [31]	SRCNN [20]	CSC [32]	ours
×2	4.78	193.26	6.82	0.88	7.54	139.03	3.21
×3	2.78	44.31	3.01	0.57	7.47	78.46	1.24
×4	1.63	-	1.96	0.42	6.39	48.24	0.75

### 3.3 Effect of IBP

Combined with iterative back projection (IBP), the denoised LR patches are applied to improve SR performance in our algorithm. According to [47], IBP has an important role to improve SR performance. But if the input is a noisy image, the model of IBP will propagate the noise to the HR image. Experimental results show that if we use IBP algorithm directly on the input LR image, the performance will become worse. The results are listed in Table 5. The iteration number of IBP here is 20. From this comparison, we can see that the superiority of our method is obvious. Other datasets Set14 and B100 can also achieve similar results. The results of Set14 and B100 are shown in S1 Table.

**Table 5:** Effect of IBP on average PSNR(dB) and SSIM (Set 5).

Scale	IBP	*σ* = 5		*σ* = 10		*σ* = 15		*σ* = 20	
		PSNR	SSIM	PSNR	SSIM	PSNR	SSIM	PSNR	SSIM
×2	×	31.48	0.831	27.76	0.665	25.03	0.531	22.93	0.432
	√	29.93	0.753	25.16	0.526	21.95	0.383	19.58	0.293
	ours	32.49	0.873	29.69	0.800	27.92	0.741	26.67	0.691
×3	×	29.19	0.801	26.59	0.660	24.33	0.537	22.47	0.442
	√	28.39	0.730	24.52	0.523	21.62	0.385	19.39	0.296
	ours	29.72	0.828	27.74	0.756	26.24	0.693	25.07	0.638
×4	×	27.65	0.765	25.58	0.646	23.64	0.537	21.97	0.449
	√	27.19	0.706	23.93	0.524	21.28	0.392	19.17	0.303
	ours	28.10	0.783	26.48	0.718	25.16	0.663	24.11	0.615

### 3.4 Effect of distance penalty

**Fig 9:**
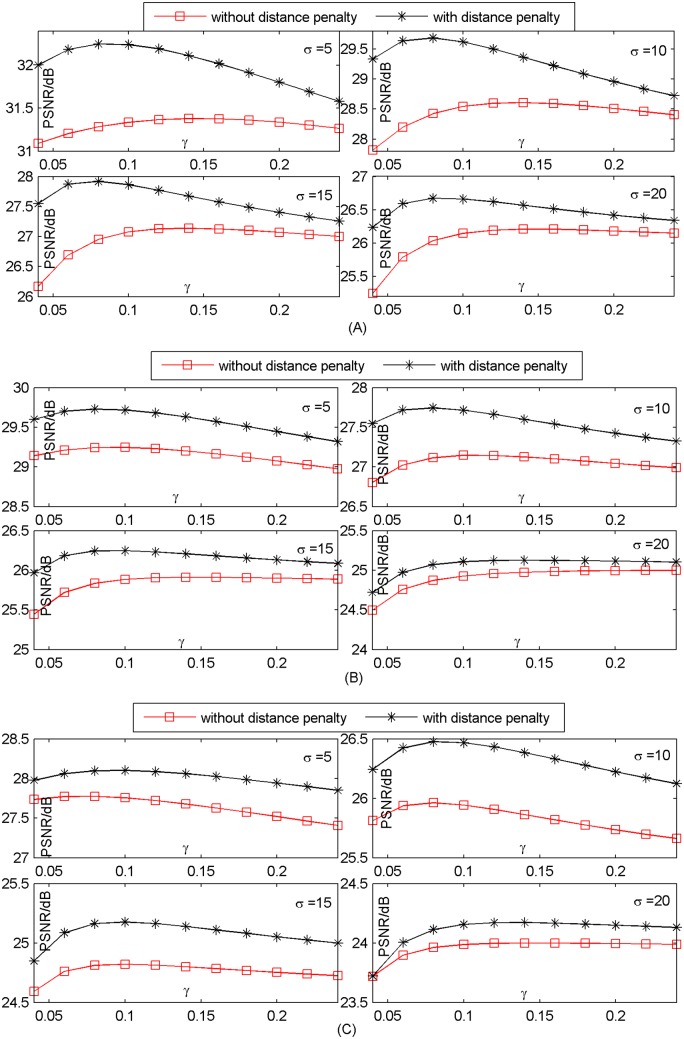
Effect of distance penalty on average PSNR (dB)(Set 5). (A) upscaling factor ×2; (B) upscaling factor ×3; (C) upscaling factor ×4.

Distance penalty is applied to model the weight. To check the effect of the penalty for improving SR performance, we perform our method with and without the penalty respectively on Set5 database. The experiments are done in different *γ*. The results are shown in in Fig 9. We can see that our method with distance penalty obtains better performance and the superiority of our method with distance penalty is obvious. Other datasets Set14 and B100 can also achieve similar results. The results of Set14 and B100 are shown in S19–S24 Figs.

## 4 Conclusion

In this research, we proposed an algorithm of noisy image super-resolution based on sparse representation. For the problem of noisy image super-resolution, most of the existing methods will become less effective because they assume that the input LR image is noise-free. The proposed algorithm can achieve simultaneously image super-resolution and denoising. For different input LR images, even if the noise variance varies, the dictionary pair does not need to be retained. The core idea of the proposed algorithm is that HR image patch is reconstructed through weighted average of similar HR example patches. In particular, atoms of learned sparse dictionary are used to compute the weight and reconstruct HR patch instead of example patches. This strategy can reduce time computation and suppress noise. In addition, LR example patches subtracted mean pixel value are also used to learn dictionary rather than just their gradient features, which will help IBP to further improve the SR performance. The experimental results show that our method performs better noise robustness.

## Supporting information

S1 Fig *γ* versus average PSNR on Set14. (upscaling factor ×2). (TIF)

S2 Fig *γ* versus average PSNR on Set14. (upscaling factor ×3). (TIF)

S3 Fig *γ* versus average PSNR on Set14. (upscaling factor ×4). (TIF)

S4 Fig *γ* versus average PSNR on B100. (upscaling factor ×2). (TIF)

S5 Fig *γ* versus average PSNR on B100. (upscaling factor ×3). (TIF)

S6 Fig *γ* versus average PSNR on B100. (upscaling factor ×4). (TIF)

S7 Fig Dictionary size influence on performance on average on Set14. (upscaling factor ×2). (TIF)

S8 Fig Dictionary size influence on performance on average on Set14. (upscaling factor ×3). (TIF)

S9 Fig Dictionary size influence on performance on average on Set14. (upscaling factor ×4). (TIF)

S10 Fig Dictionary size influence on performance on average on B100. (upscaling factor ×2). (TIF)

S11 Fig Dictionary size influence on performance on average on B100. (upscaling factor ×3). (TIF)

S12 Fig Dictionary size influence on performance on average on B100. (upscaling factor ×4). (TIF)

S13 Fig Number of similar atoms influence on performance on average on Set14. (upscaling factor ×2). (TIF)

S14 Fig Number of similar atoms influence on performance on average on Set14. (upscaling factor ×3). (TIF)

S15 Fig Number of similar atoms influence on performance on average on Set14. (upscaling factor ×4). (TIF)

S16 Fig Number of similar atoms influence on performance on average on B100. (upscaling factor ×2). (TIF)

S17 Fig Number of similar atoms influence on performance on average on B100. (upscaling factor ×3). (TIF)

S18 Fig Number of similar atoms influence on performance on average on B100. (upscaling factor ×4). (TIF)

S19 Fig Effect of Distance Penalty on Average PSNR (dB) on average on Set14. (upscaling factor ×2). (TIF)

S20 Fig Effect of Distance Penalty on Average PSNR (dB) on average on Set14. (upscaling factor ×3). (TIF)

S21 Fig Effect of Distance Penalty on Average PSNR (dB) on average on Set14. (upscaling factor ×4). (TIF)

S22 Fig Effect of Distance Penalty on Average PSNR (dB) on average on B100. (upscaling factor ×2). (TIF)

S23 Fig Effect of Distance Penalty on Average PSNR (dB) on average on B100. (upscaling factor ×3). (TIF)

S24 Fig Effect of Distance Penalty on Average PSNR (dB) on average on B100. (upscaling factor ×4). (TIF)

S1 Table Effect of IBP on Average PSNR (dB) and SSIM (Set14 and B100). (PDF)
