# Supplementary figures and images for: Dictionary learning based noisy image super-resolution via distance penalty weight model
Source: PLoS One. 2017 Jul 31;12(7):e0182165. doi: 10.1371/journal.pone.0182165 (PMC5536359; doi:10.1371/journal.pone.0182165)

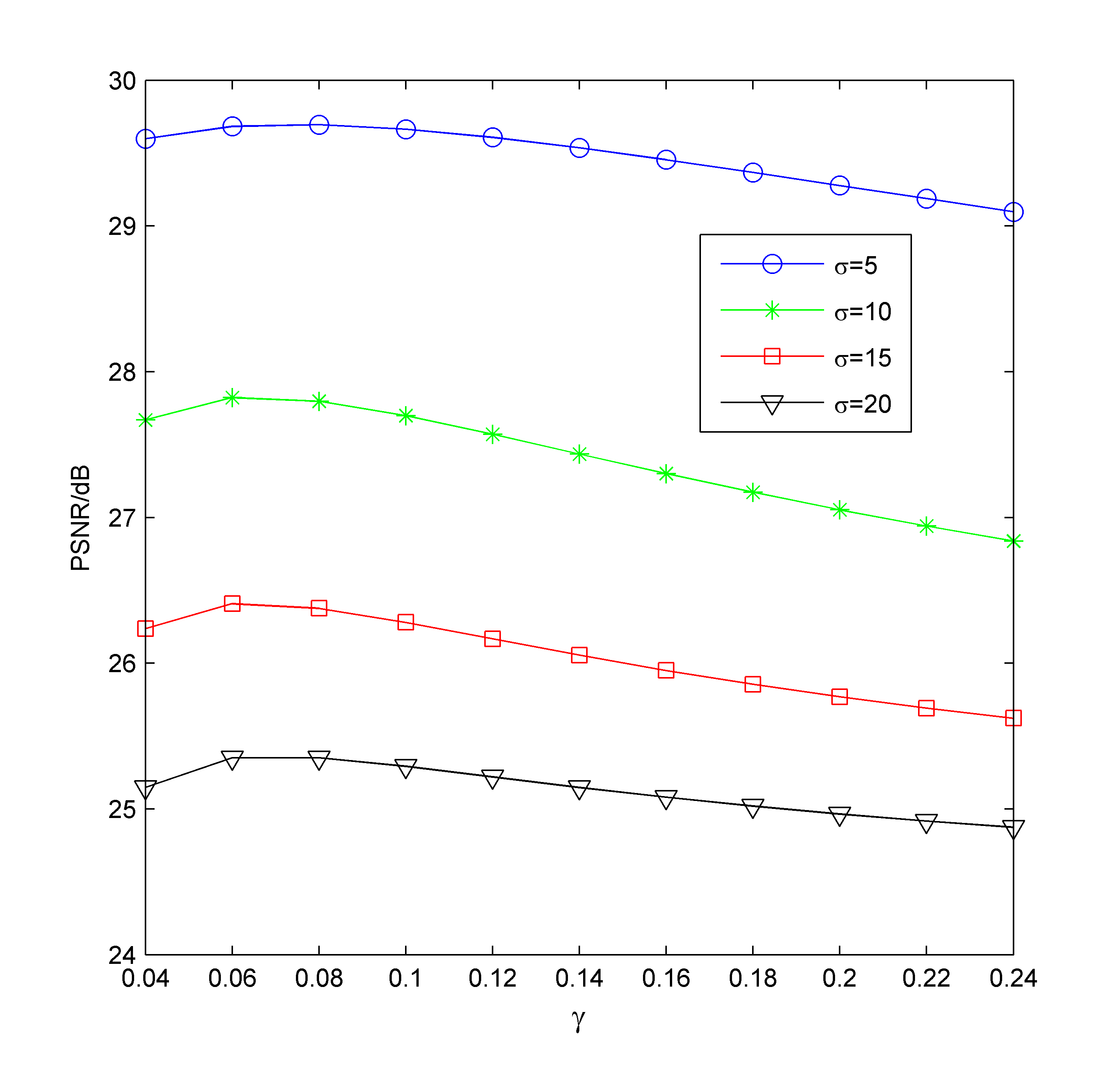

Supplement: S1 Fig — (TIF) [file pone.0182165.s001.tif]

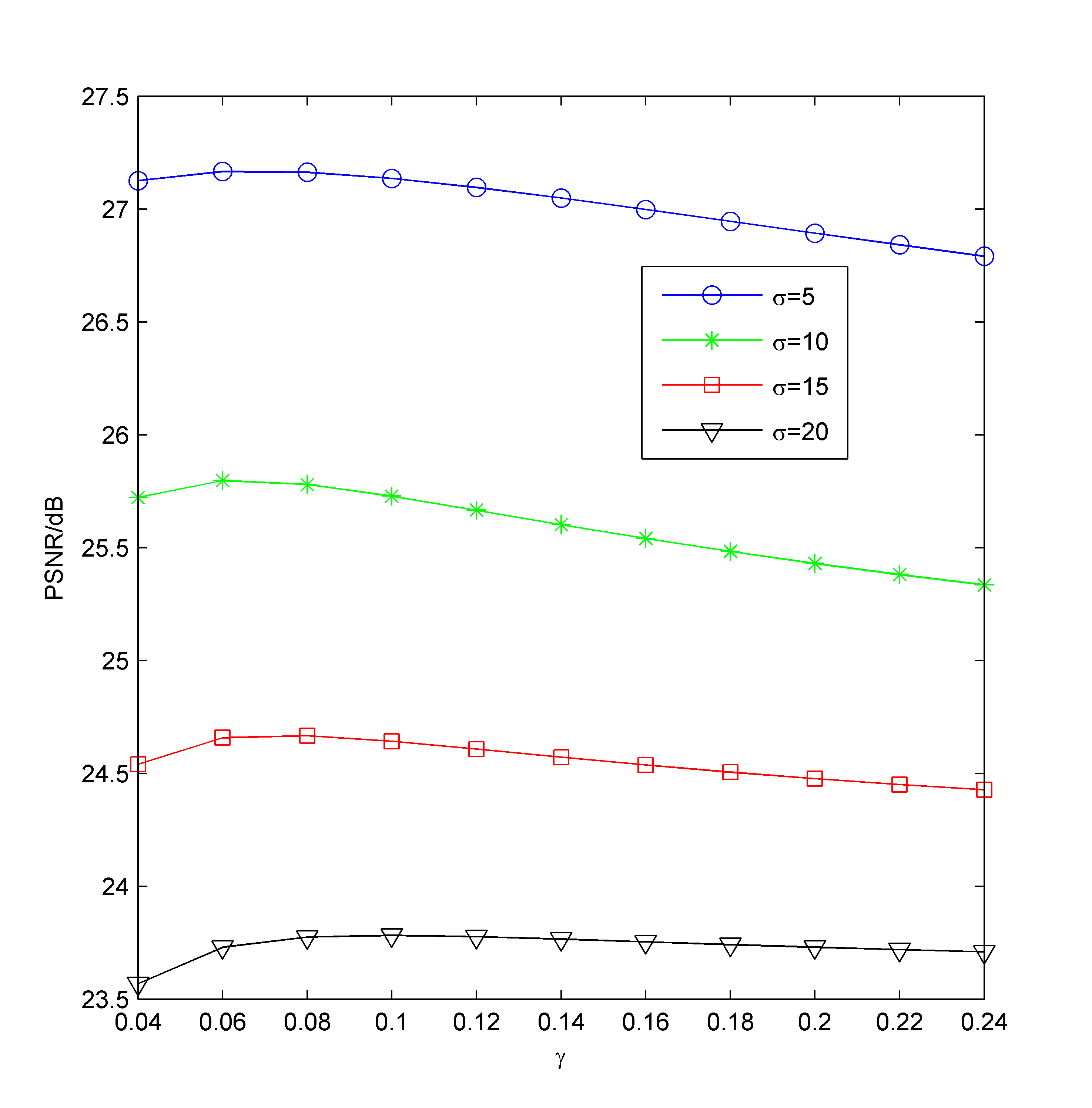

Supplement: S2 Fig — (TIF) [file pone.0182165.s002.tif]

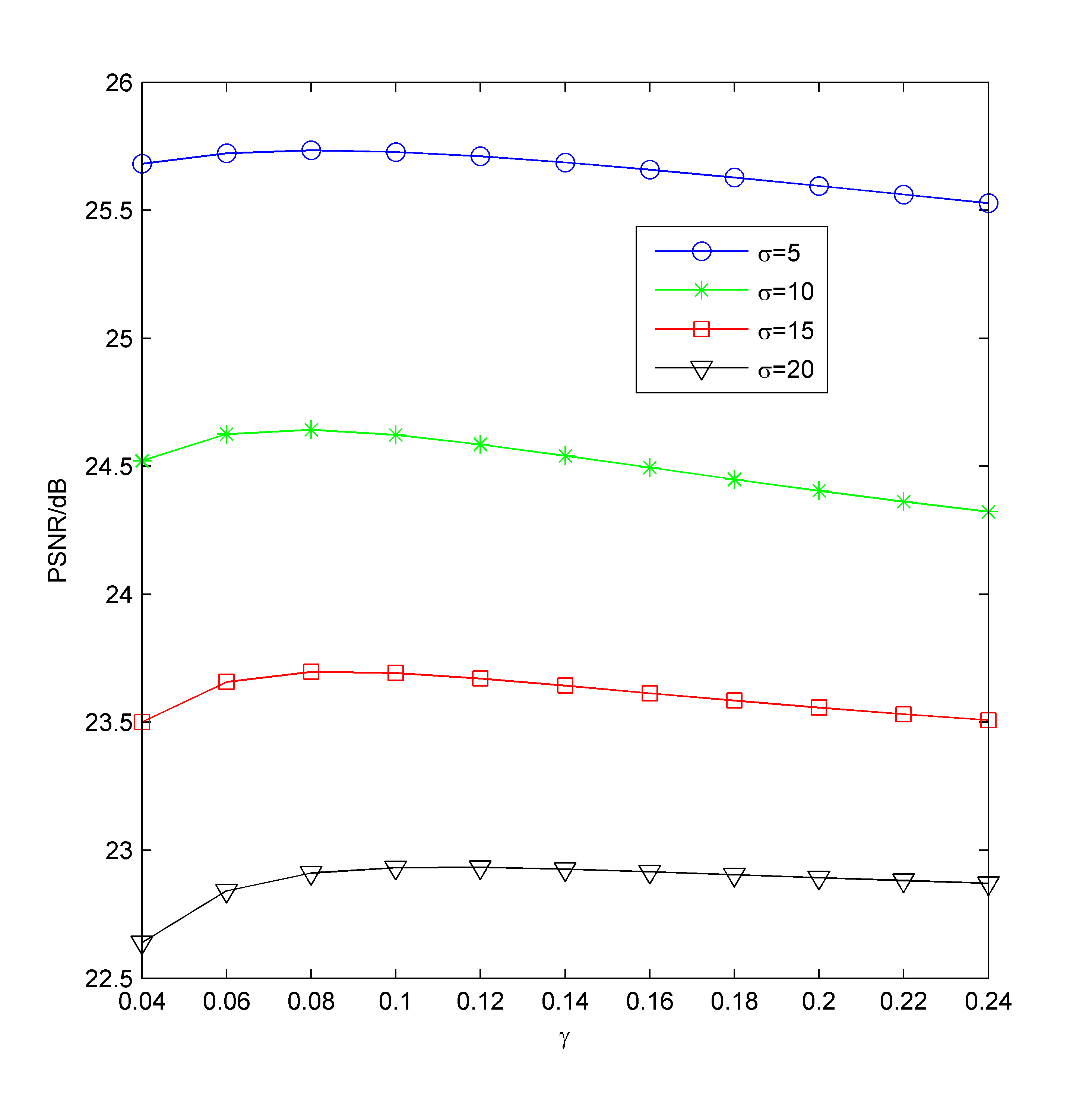

Supplement: S3 Fig — (TIF) [file pone.0182165.s003.tif]

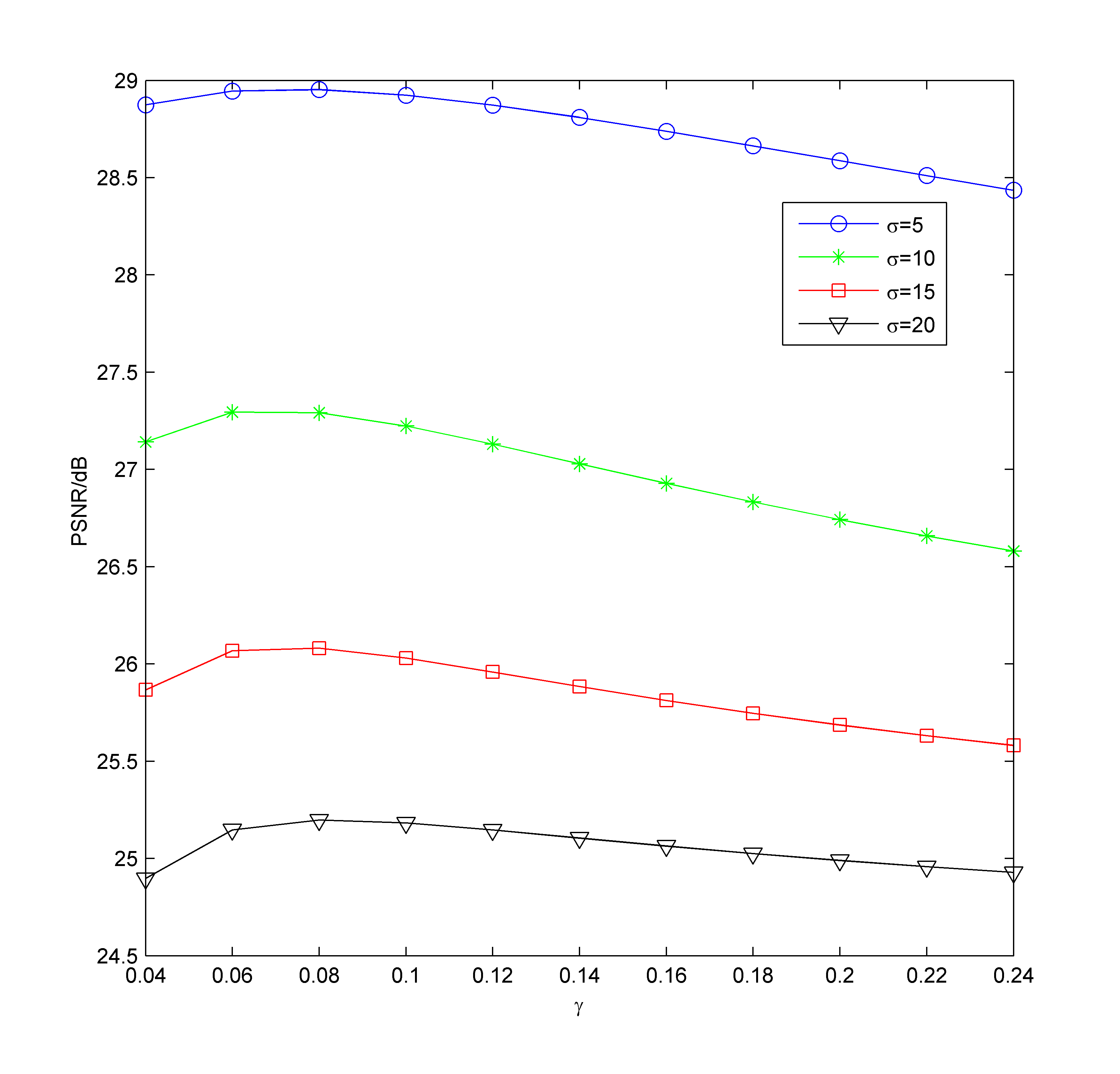

Supplement: S4 Fig — (TIF) [file pone.0182165.s004.tif]

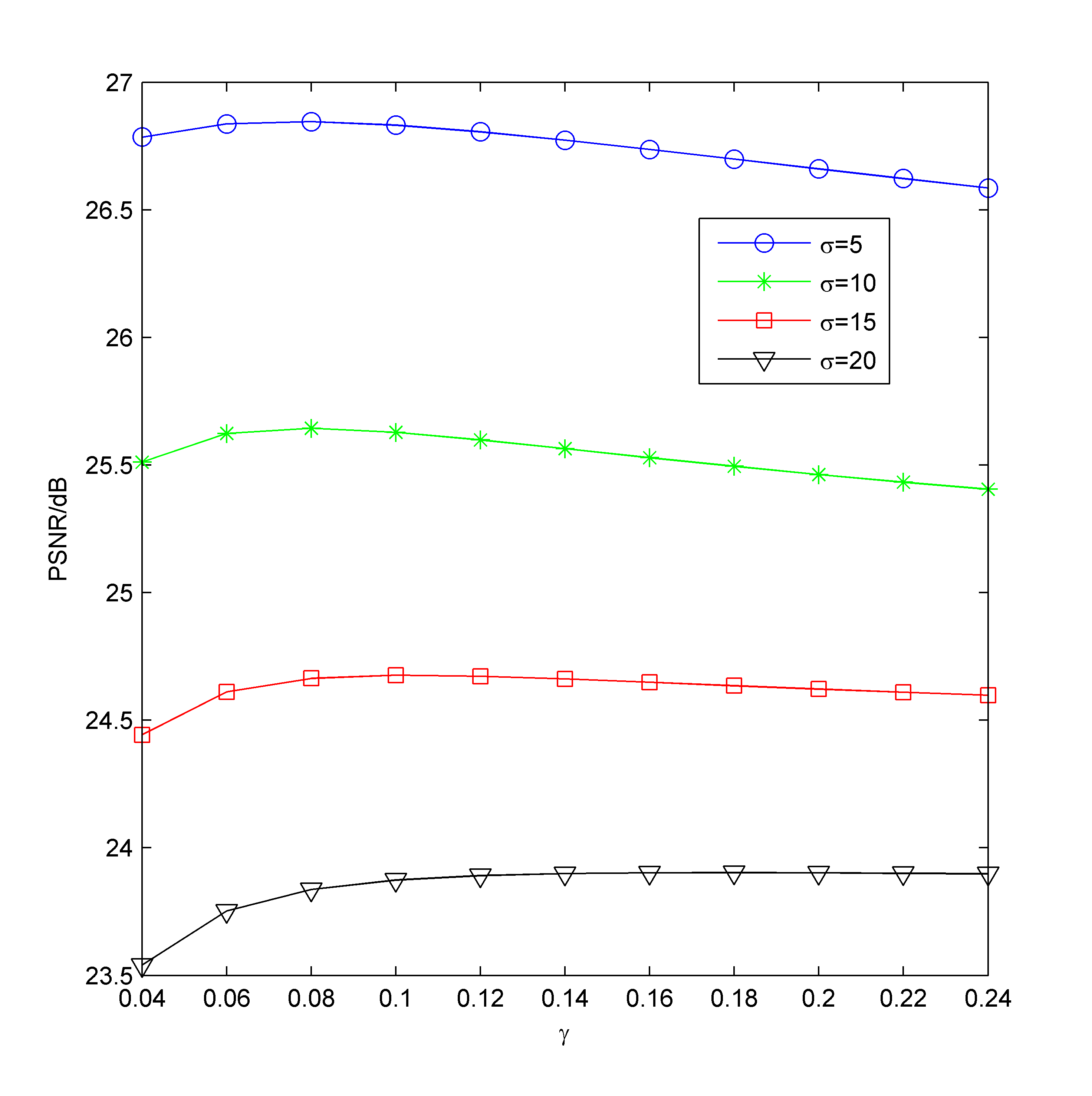

Supplement: S5 Fig — (TIF) [file pone.0182165.s005.tif]

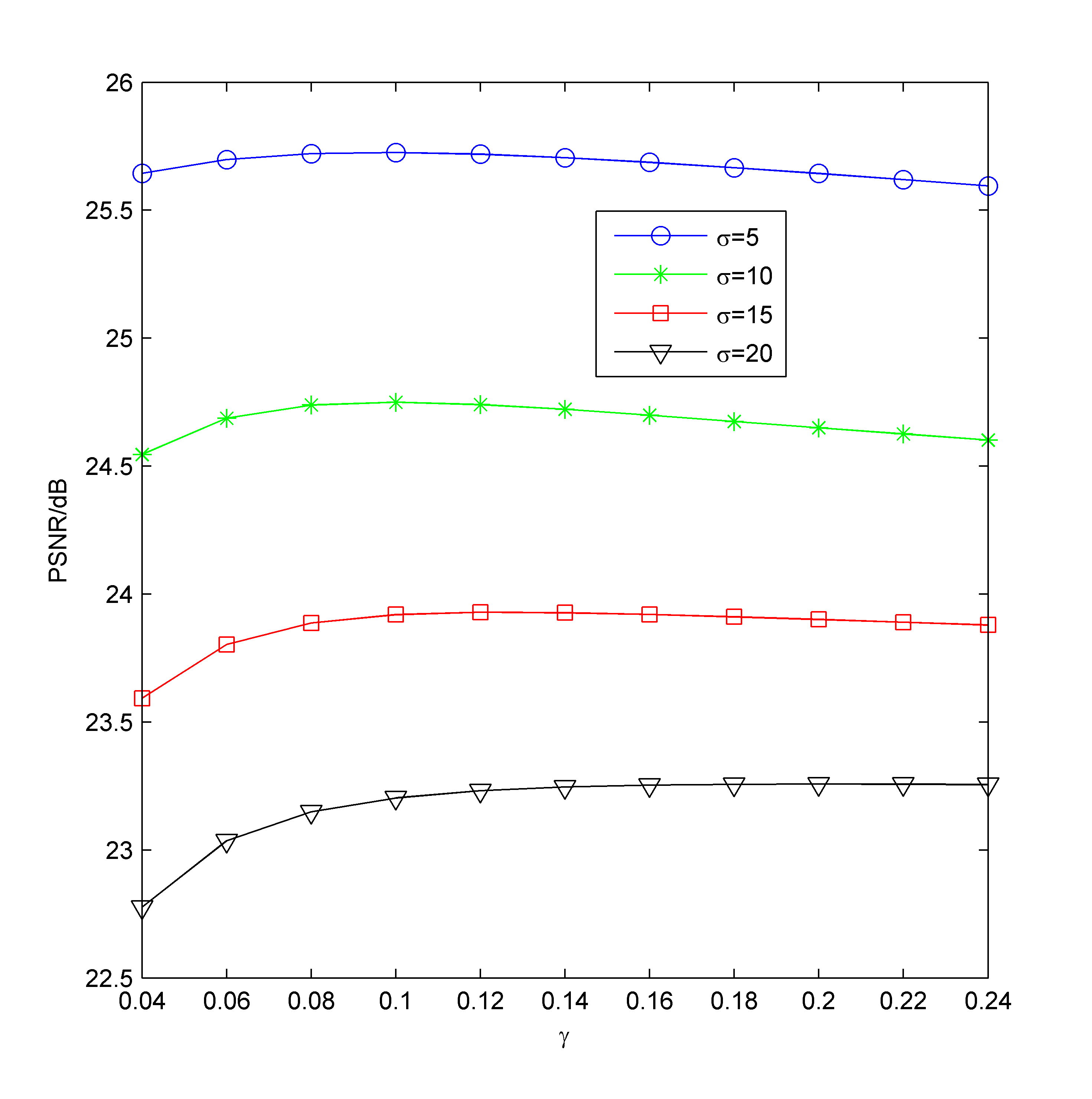

Supplement: S6 Fig — (TIF) [file pone.0182165.s006.tif]

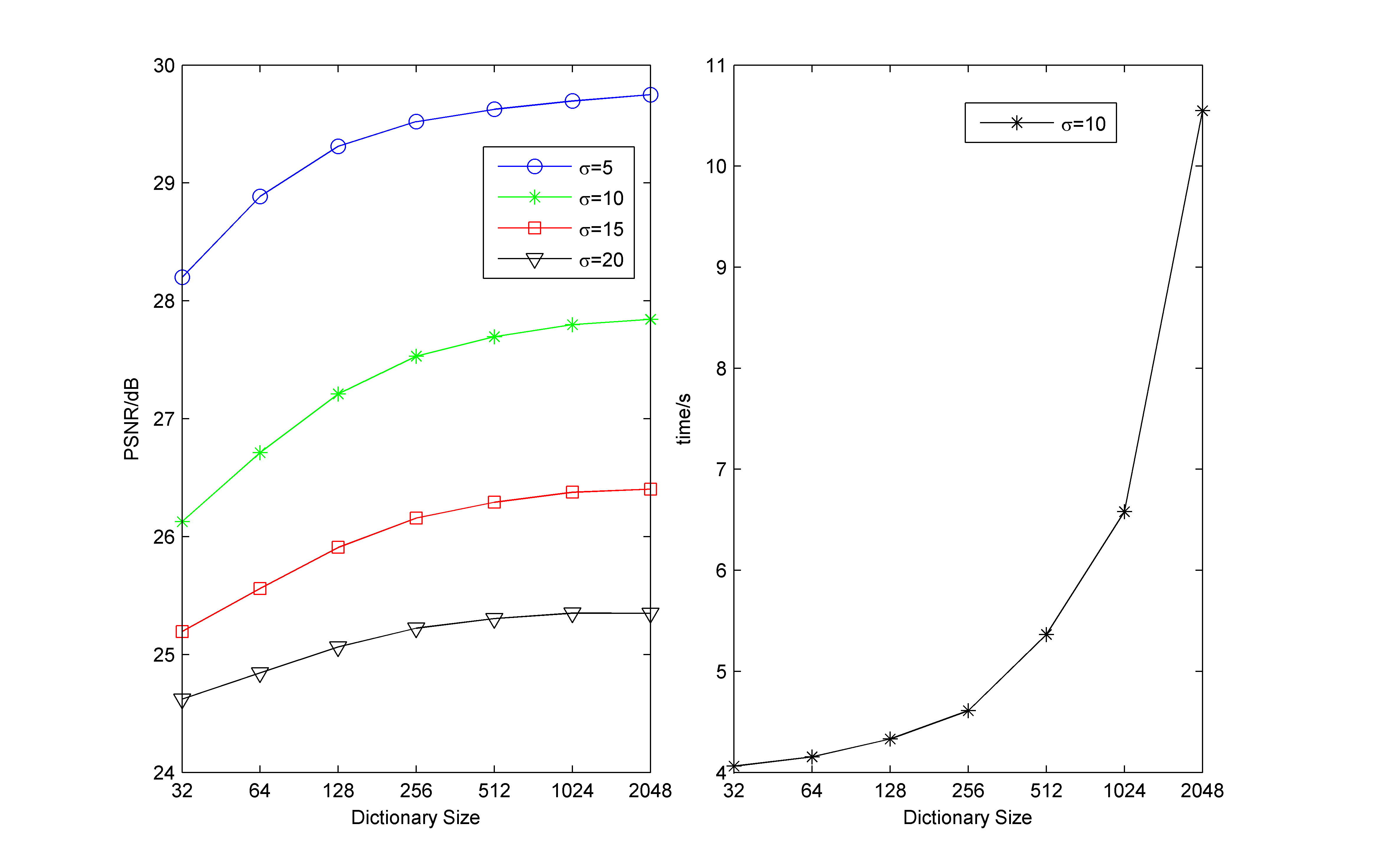

Supplement: S7 Fig — (TIF) [file pone.0182165.s007.tif]

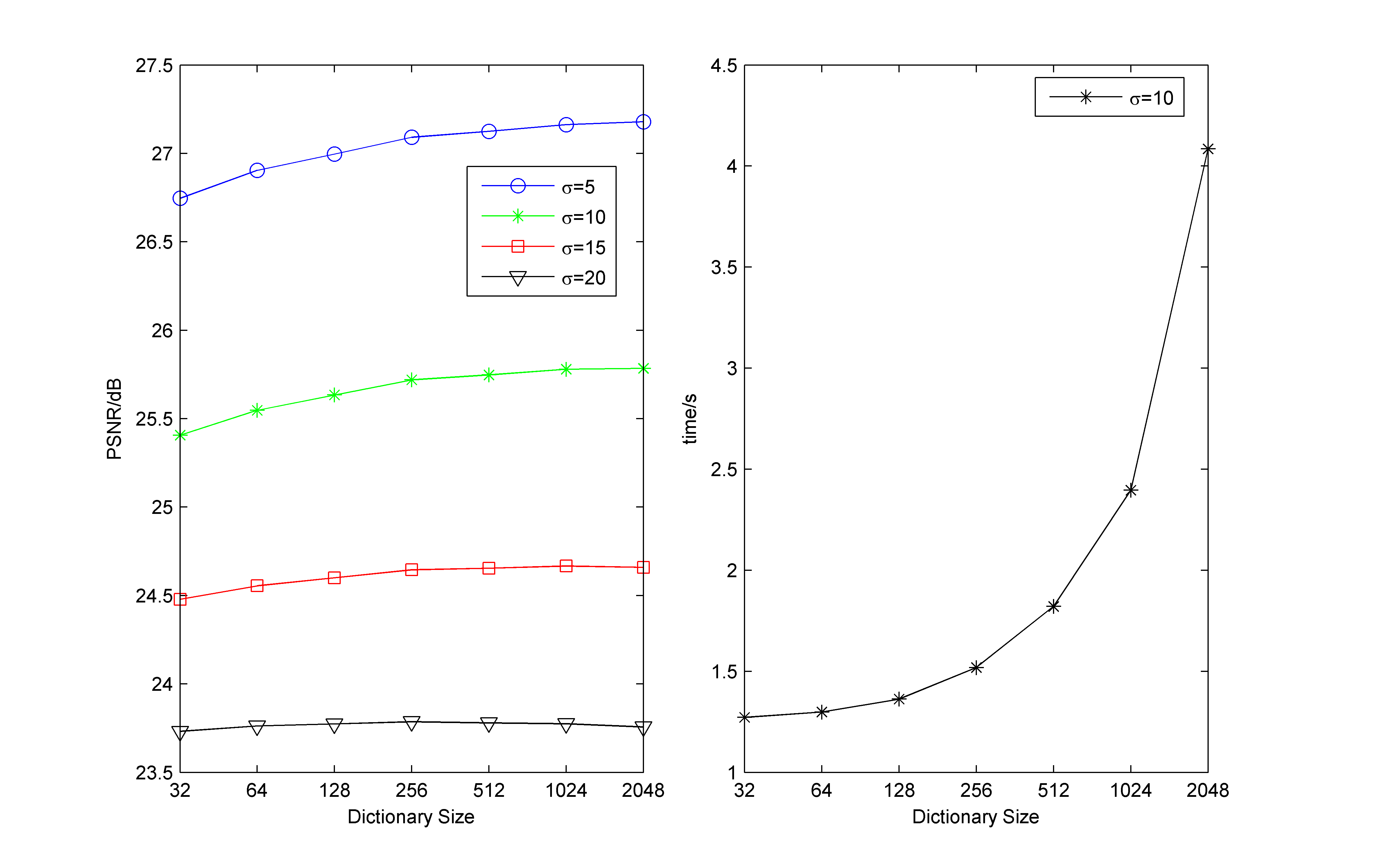

Supplement: S8 Fig — (TIF) [file pone.0182165.s008.tif]

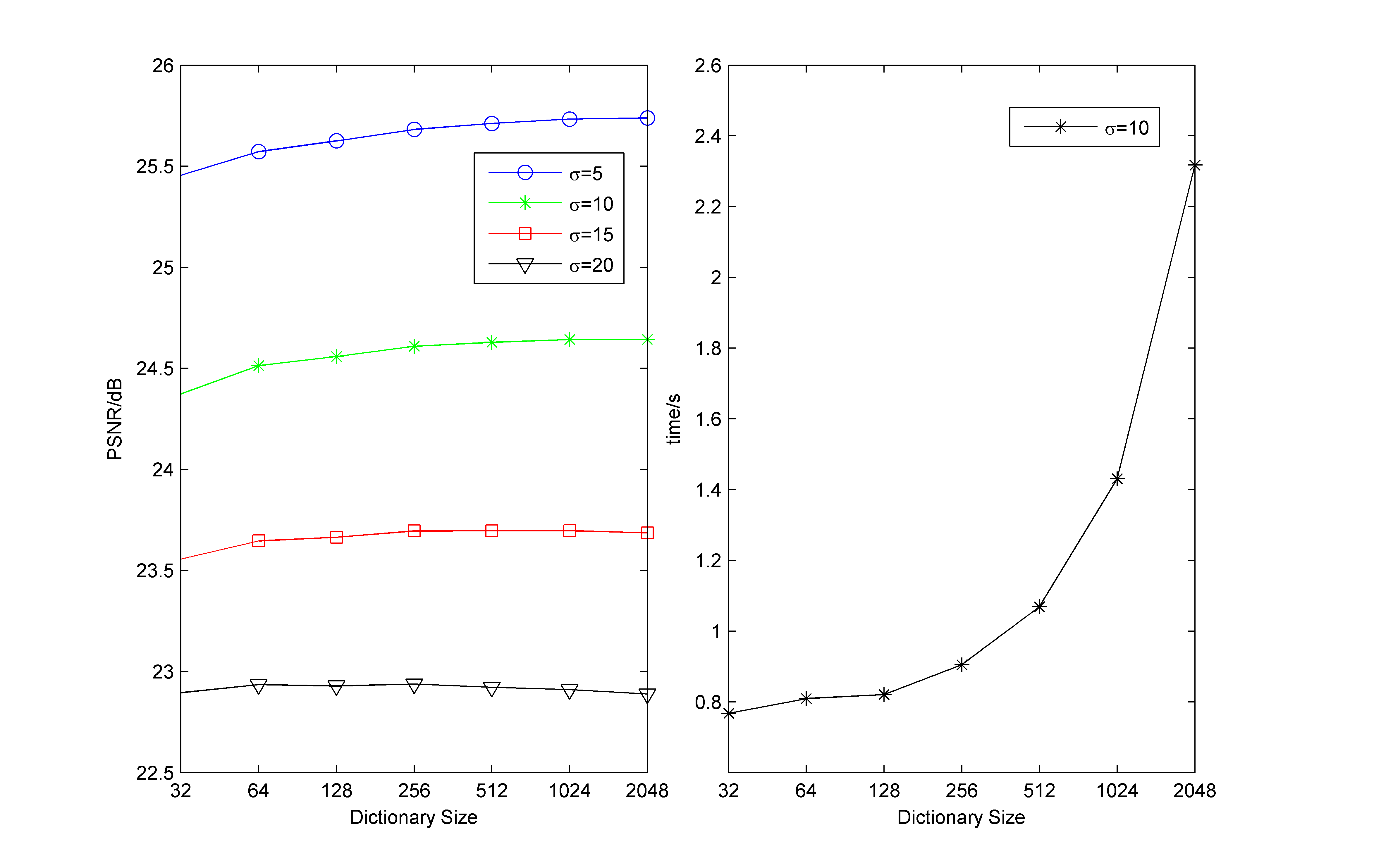

Supplement: S9 Fig — (TIF) [file pone.0182165.s009.tif]

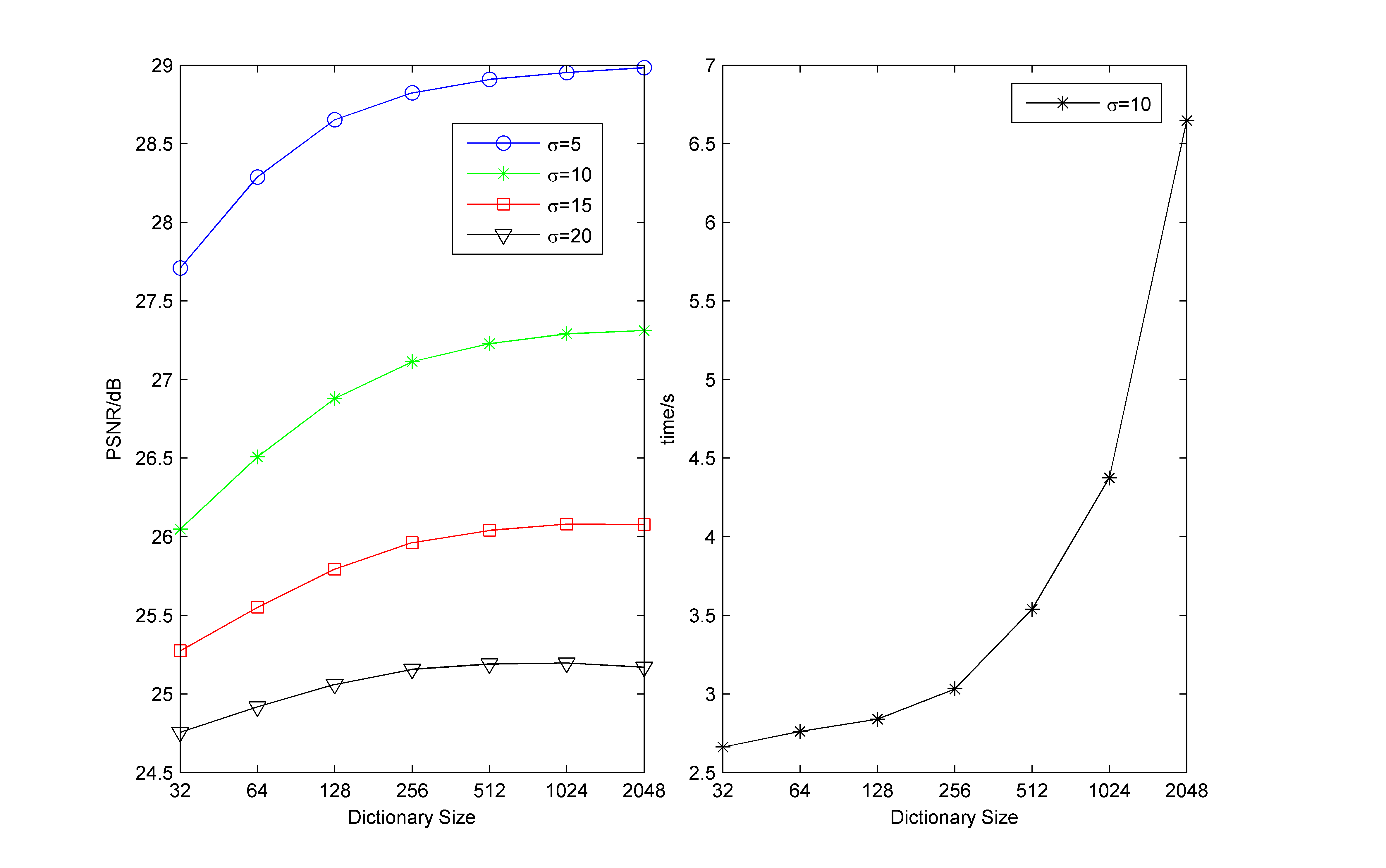

Supplement: S10 Fig — (TIF) [file pone.0182165.s010.tif]

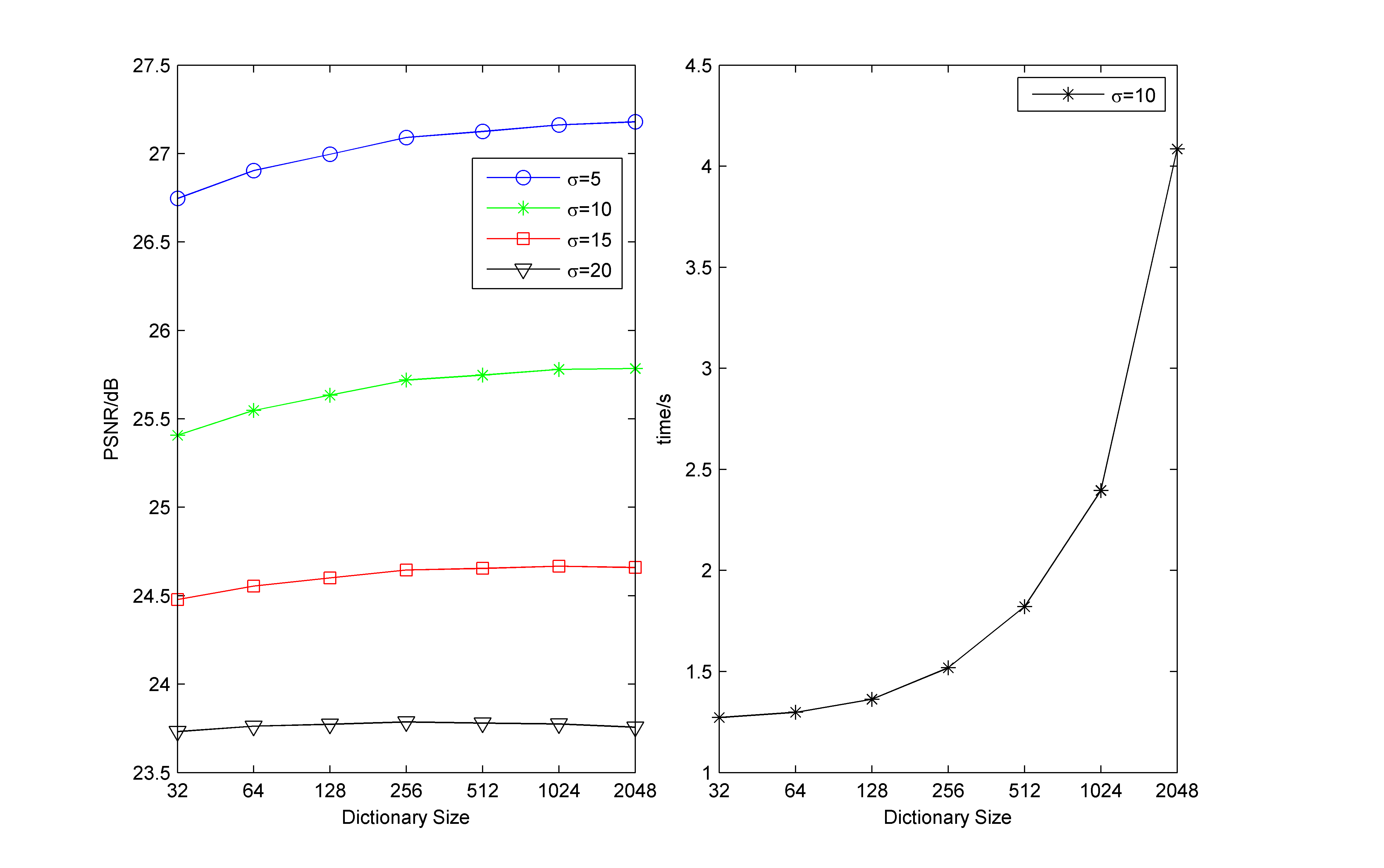

Supplement: S11 Fig — (TIF) [file pone.0182165.s011.tif]

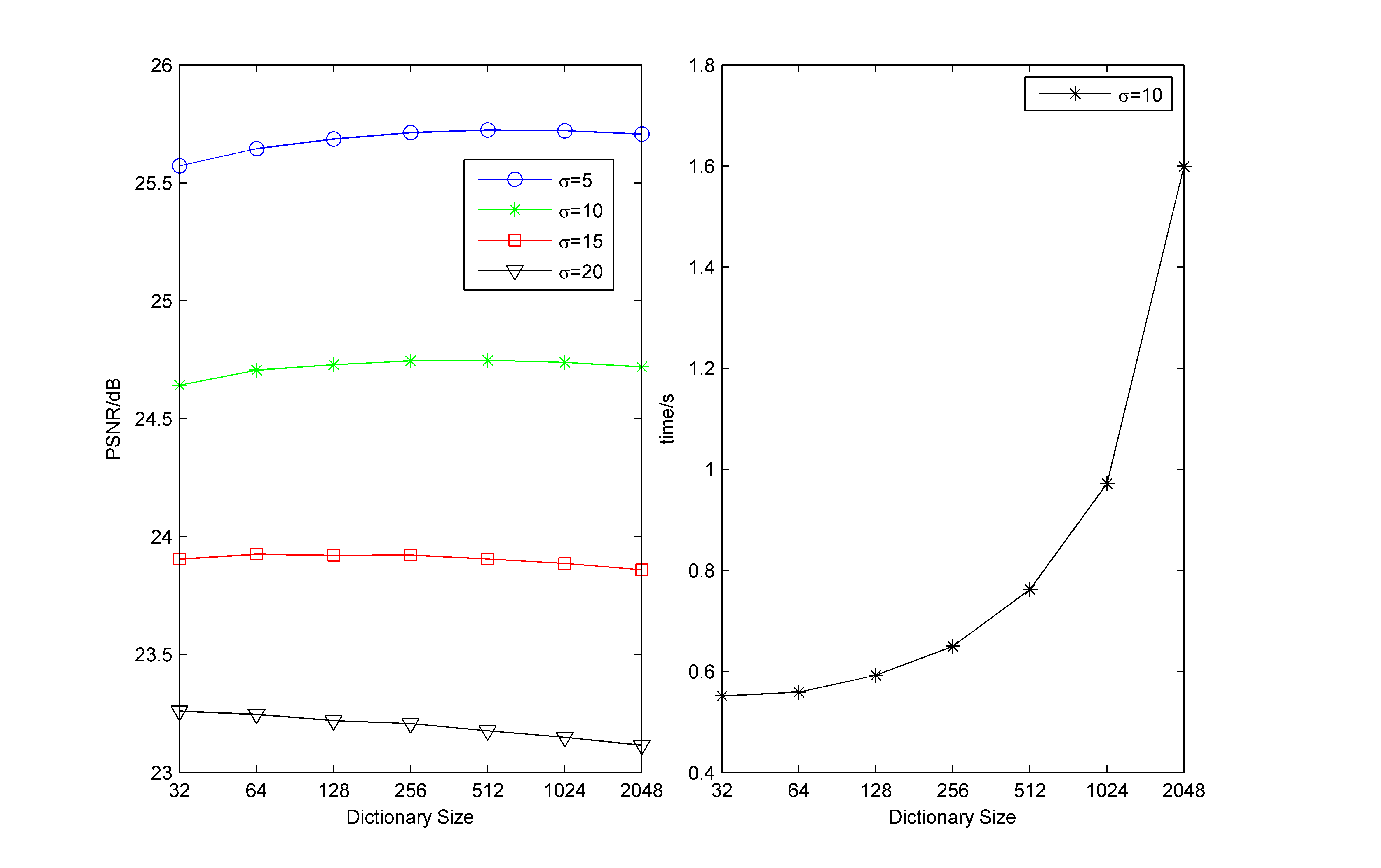

Supplement: S12 Fig — (TIF) [file pone.0182165.s012.tif]

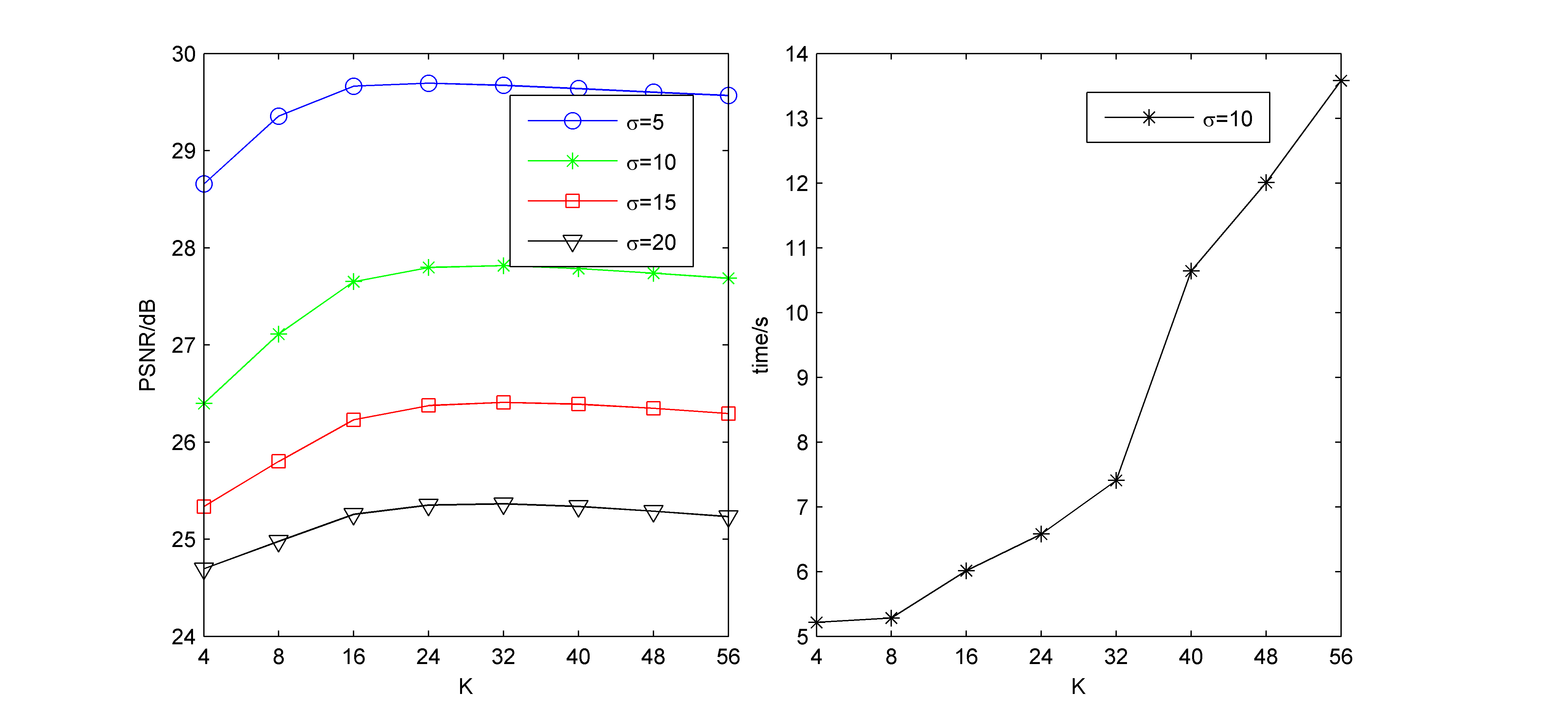

Supplement: S13 Fig — (TIF) [file pone.0182165.s013.tif]

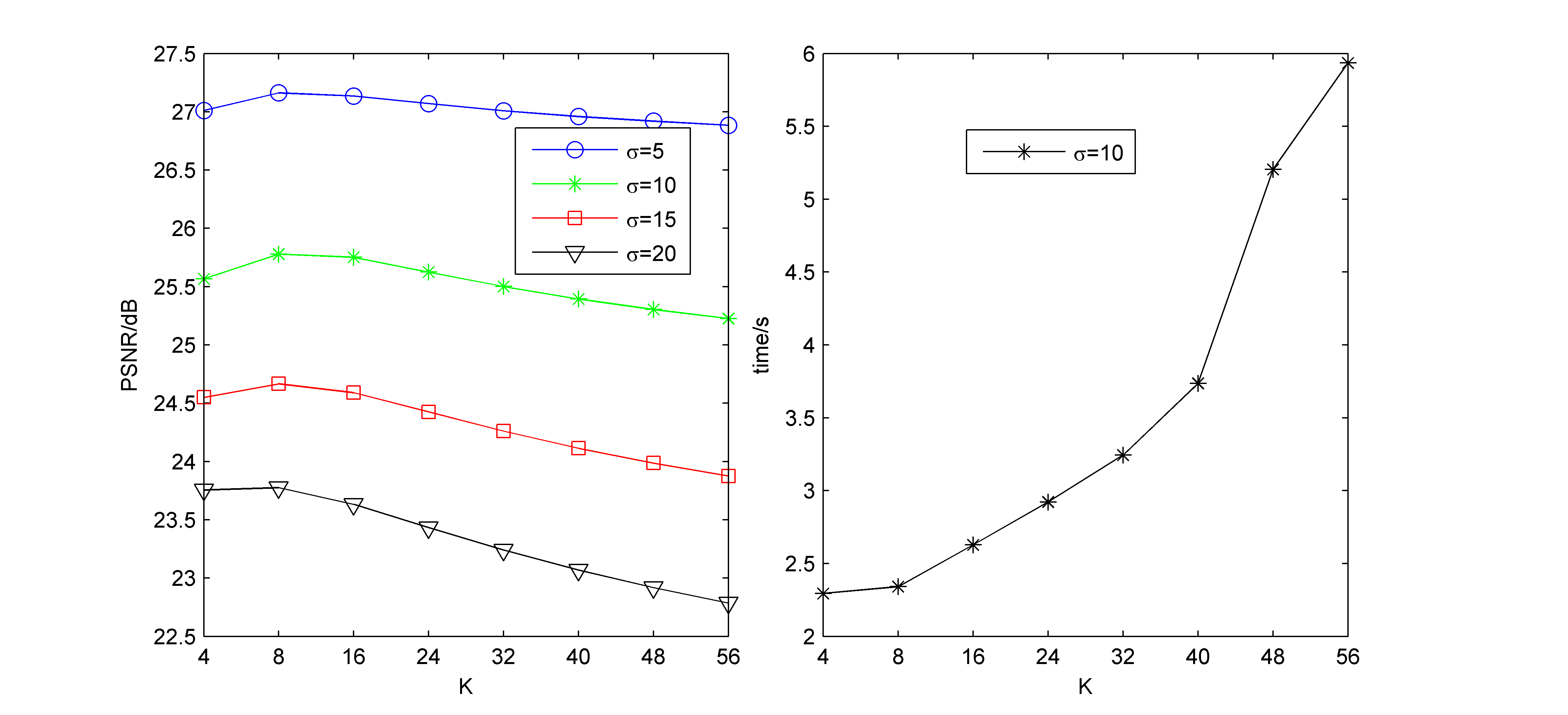

Supplement: S14 Fig — (TIF) [file pone.0182165.s014.tif]

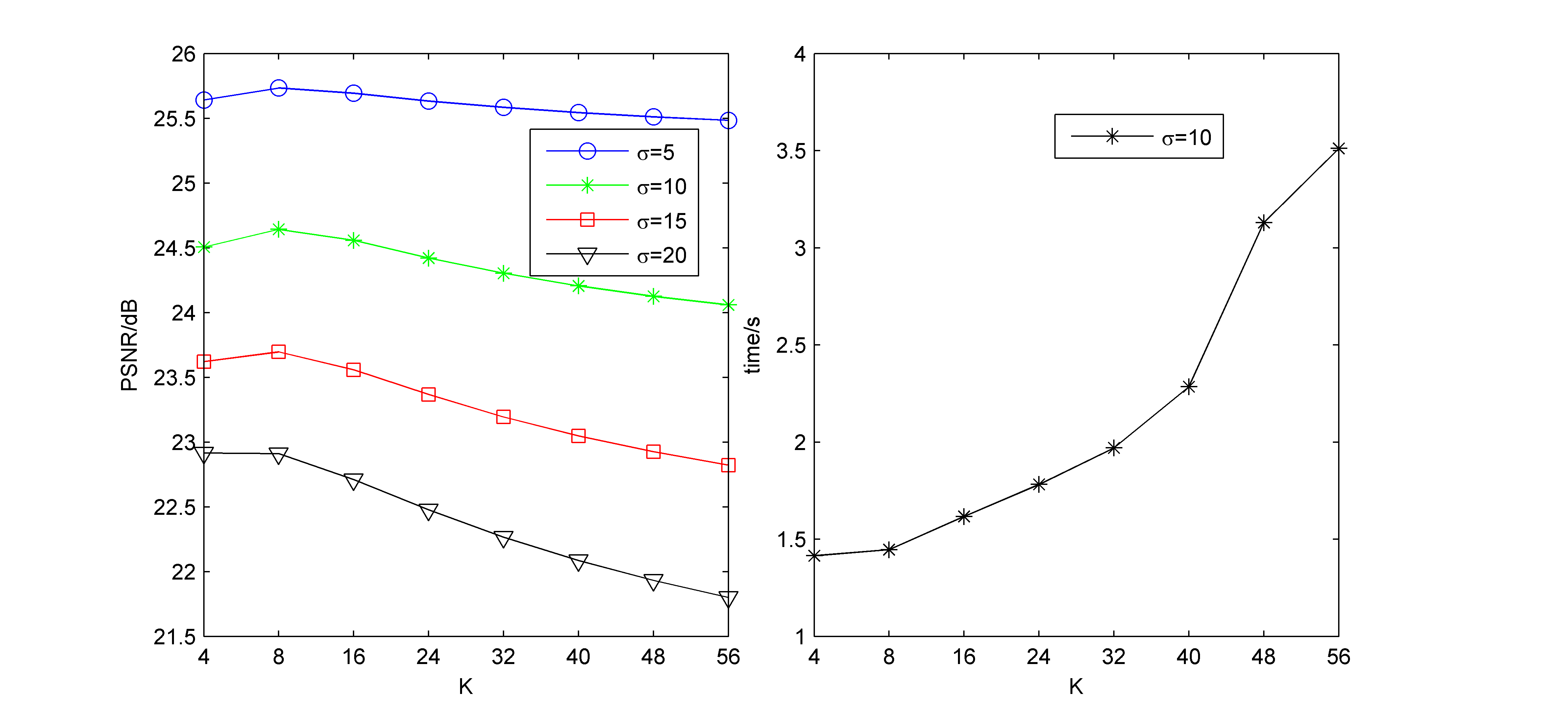

Supplement: S15 Fig — (TIF) [file pone.0182165.s015.tif]

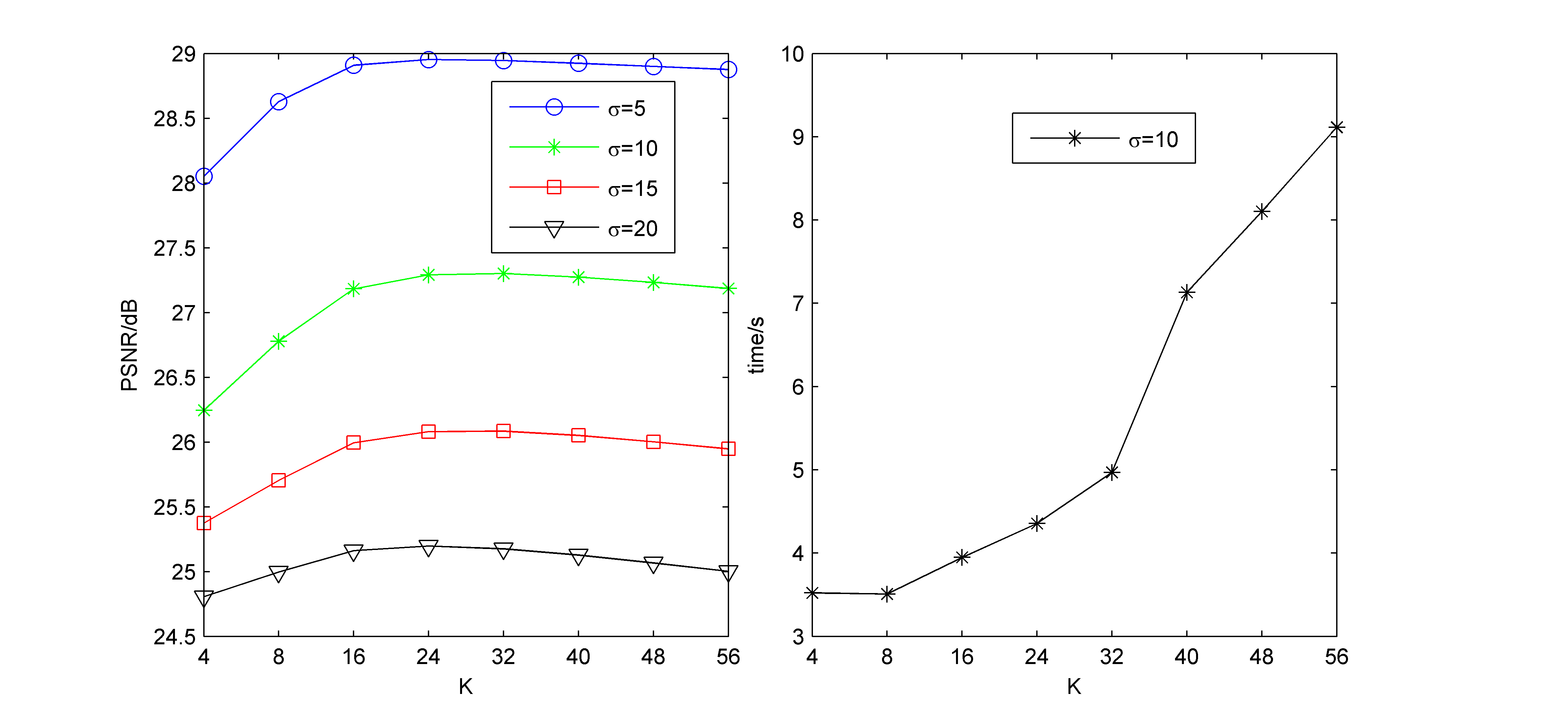

Supplement: S16 Fig — (TIF) [file pone.0182165.s016.tif]

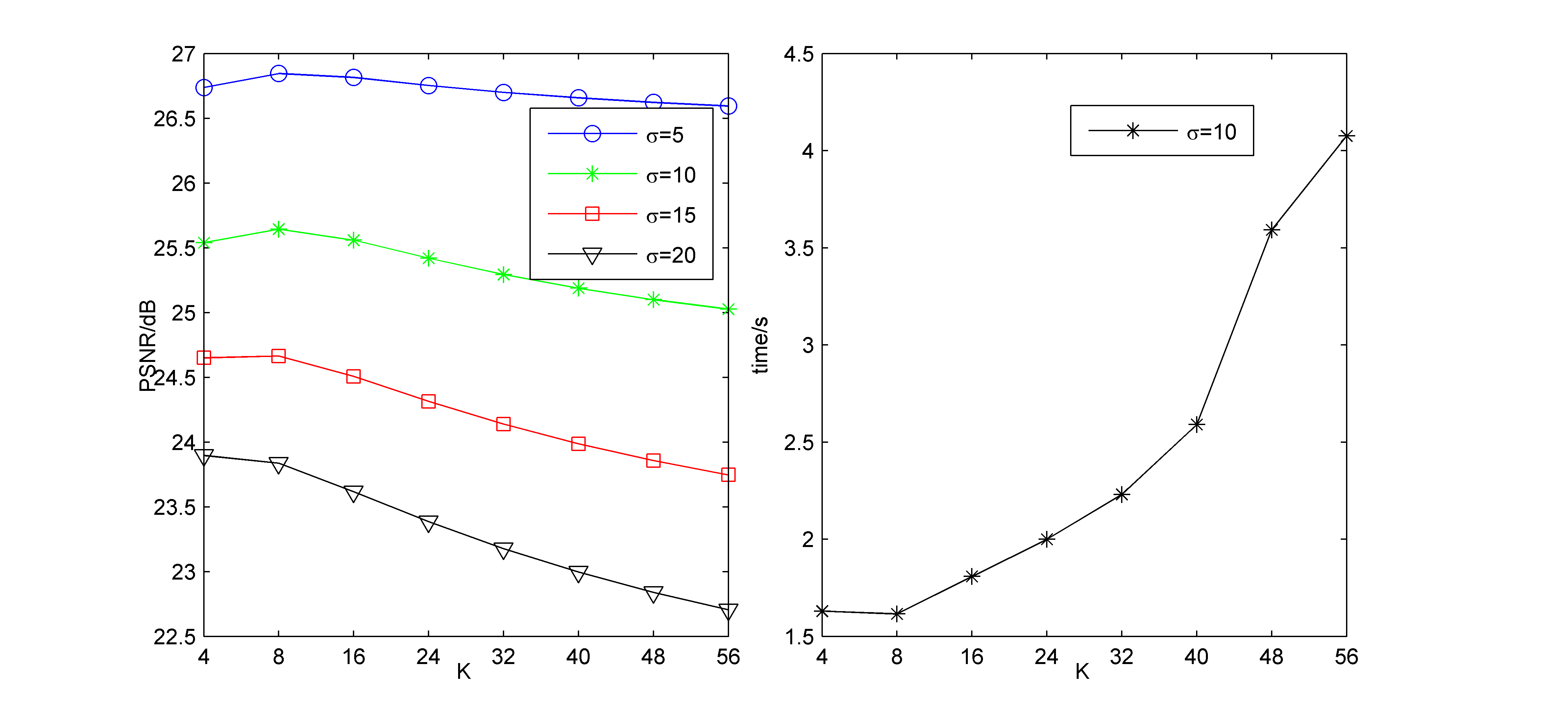

Supplement: S17 Fig — (TIF) [file pone.0182165.s017.tif]

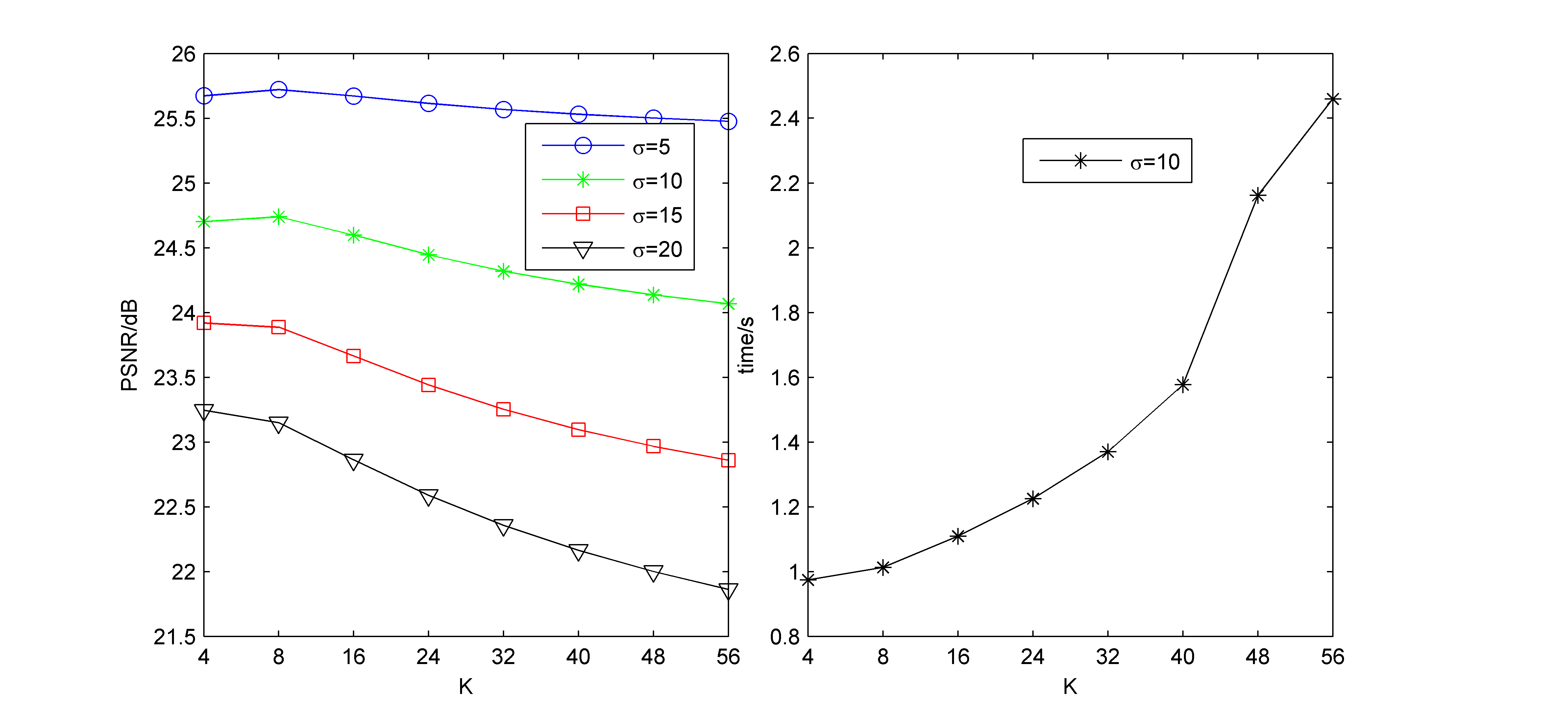

Supplement: S18 Fig — (TIF) [file pone.0182165.s018.tif]

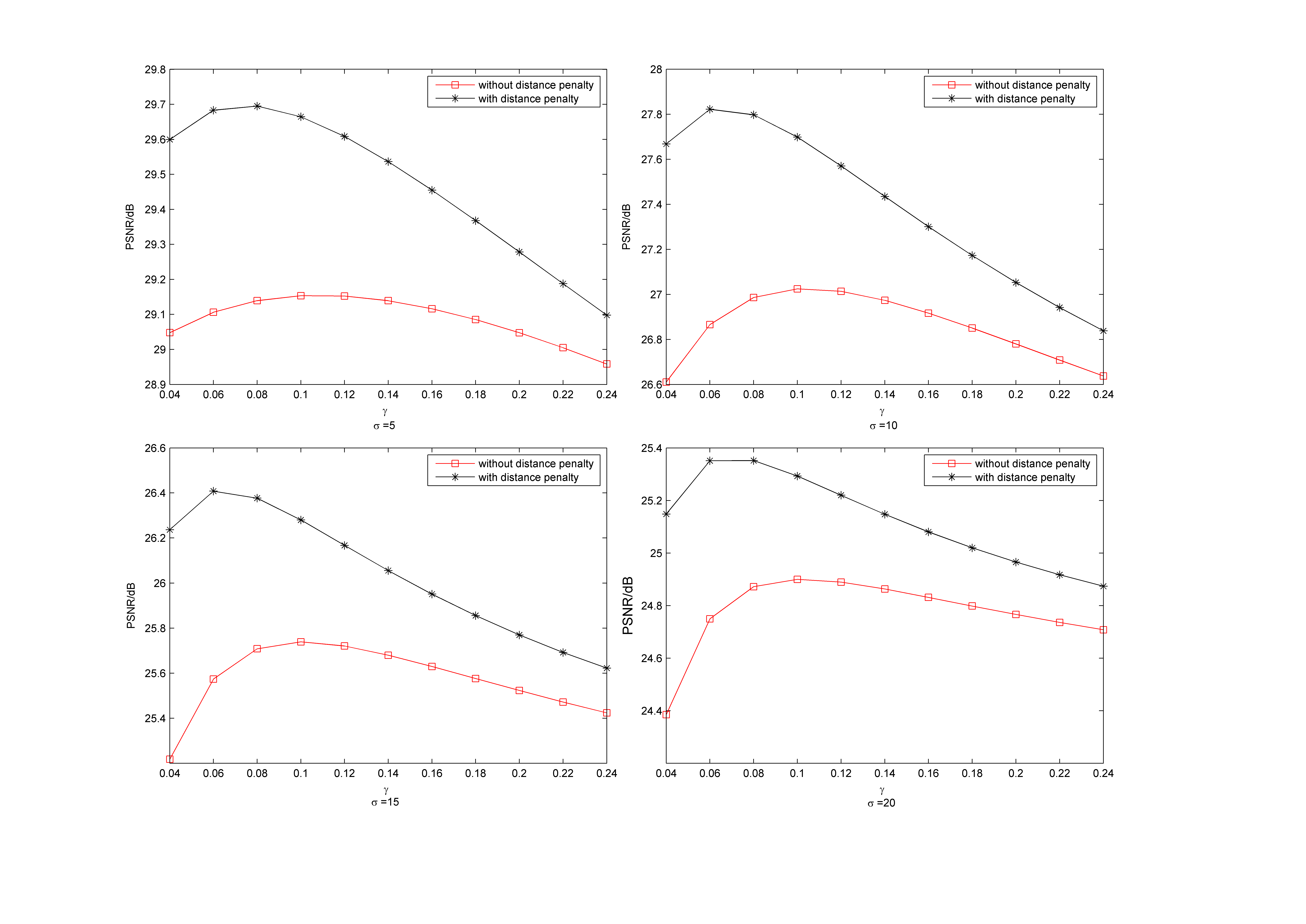

Supplement: S19 Fig — (TIF) [file pone.0182165.s019.tif]

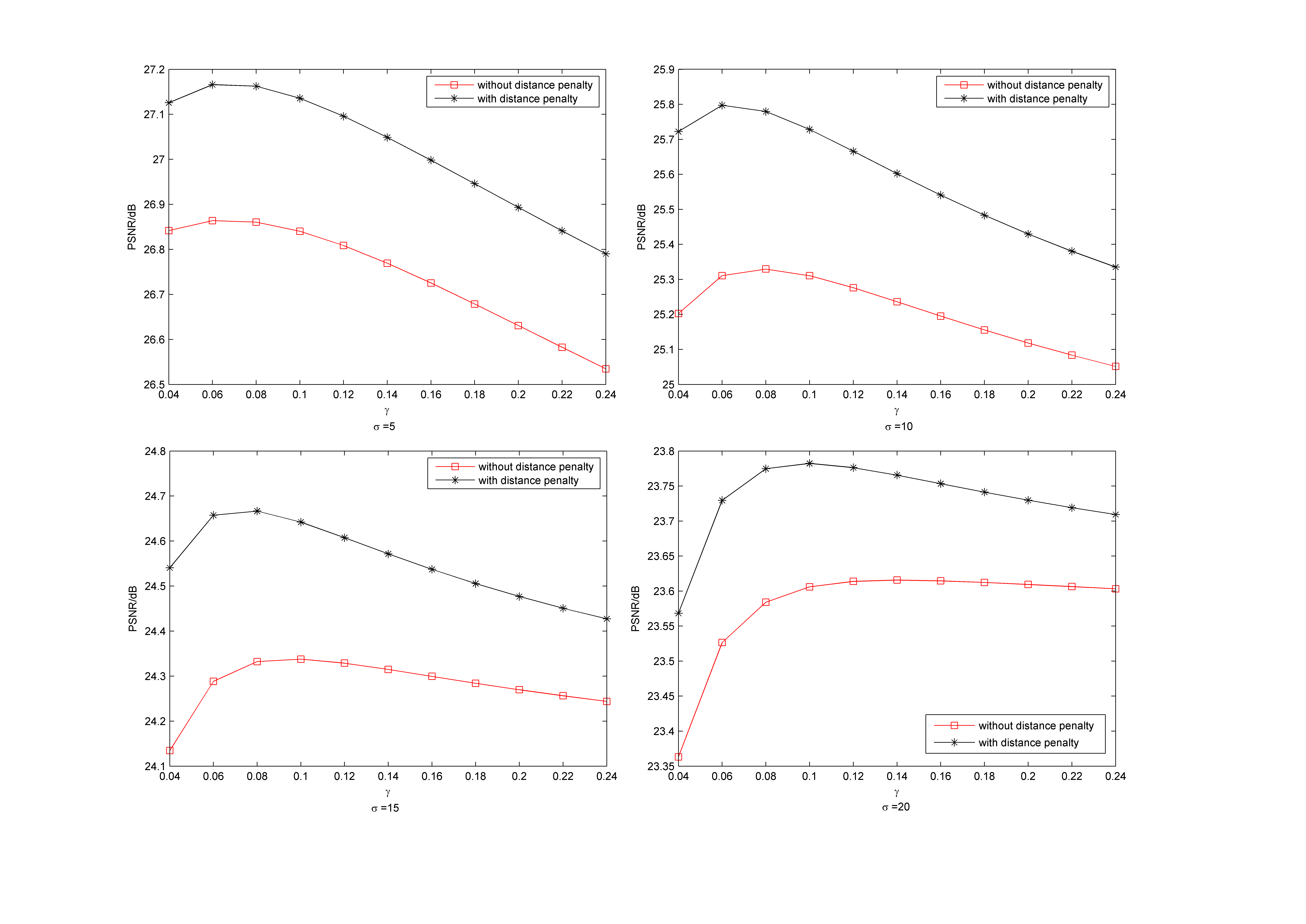

Supplement: S20 Fig — (TIF) [file pone.0182165.s020.tif]

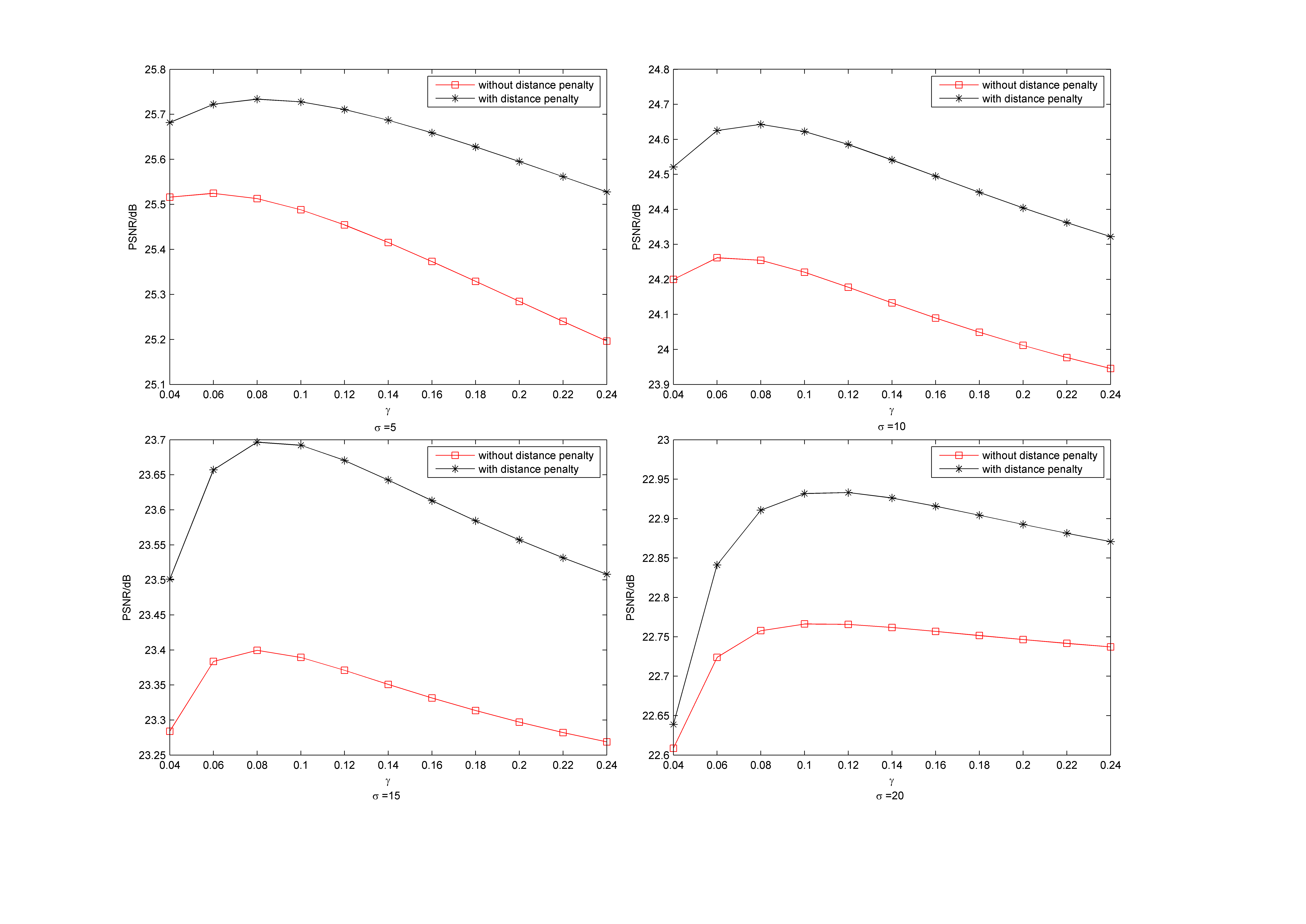

Supplement: S21 Fig — (TIF) [file pone.0182165.s021.tif]

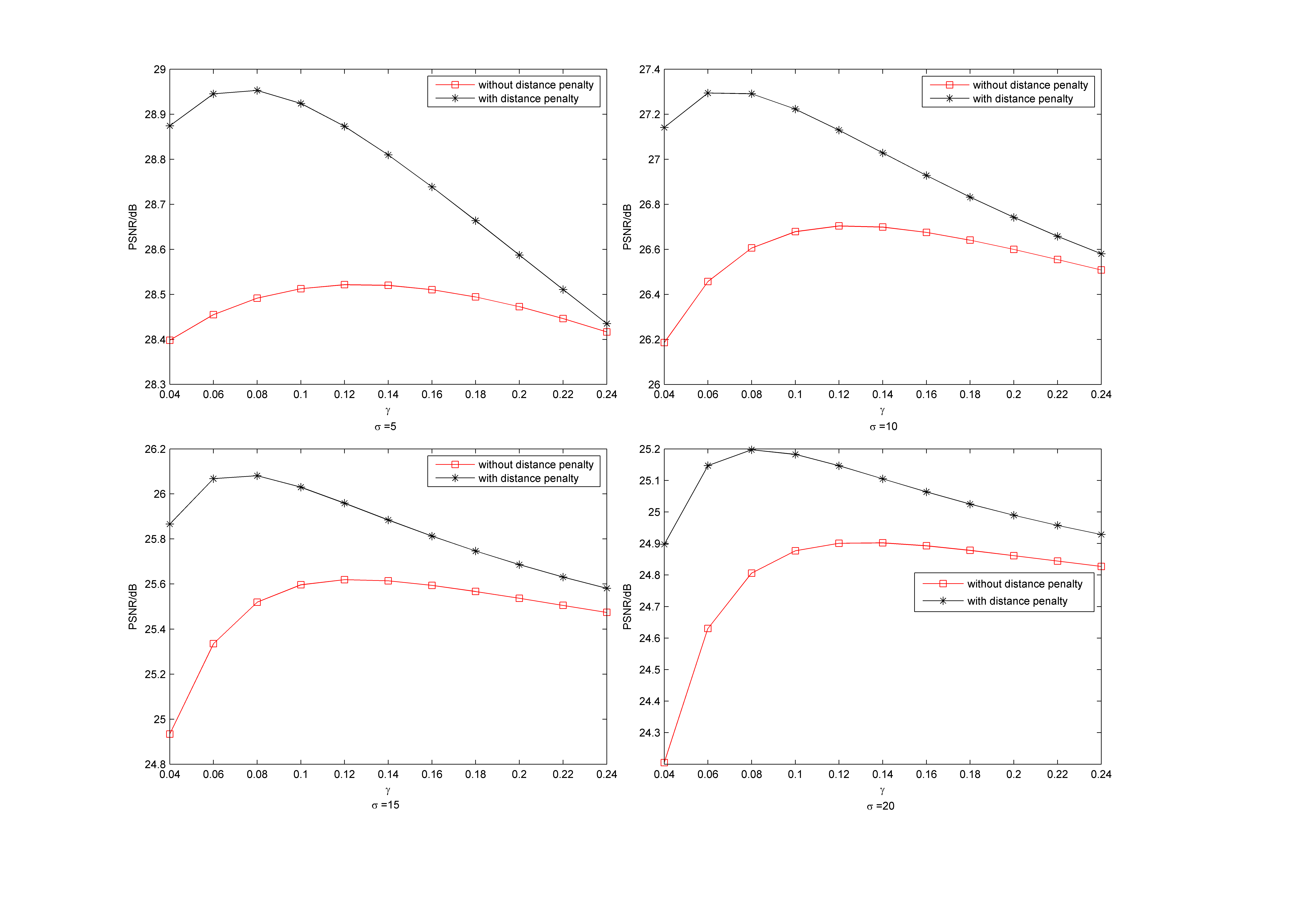

Supplement: S22 Fig — (TIF) [file pone.0182165.s022.tif]

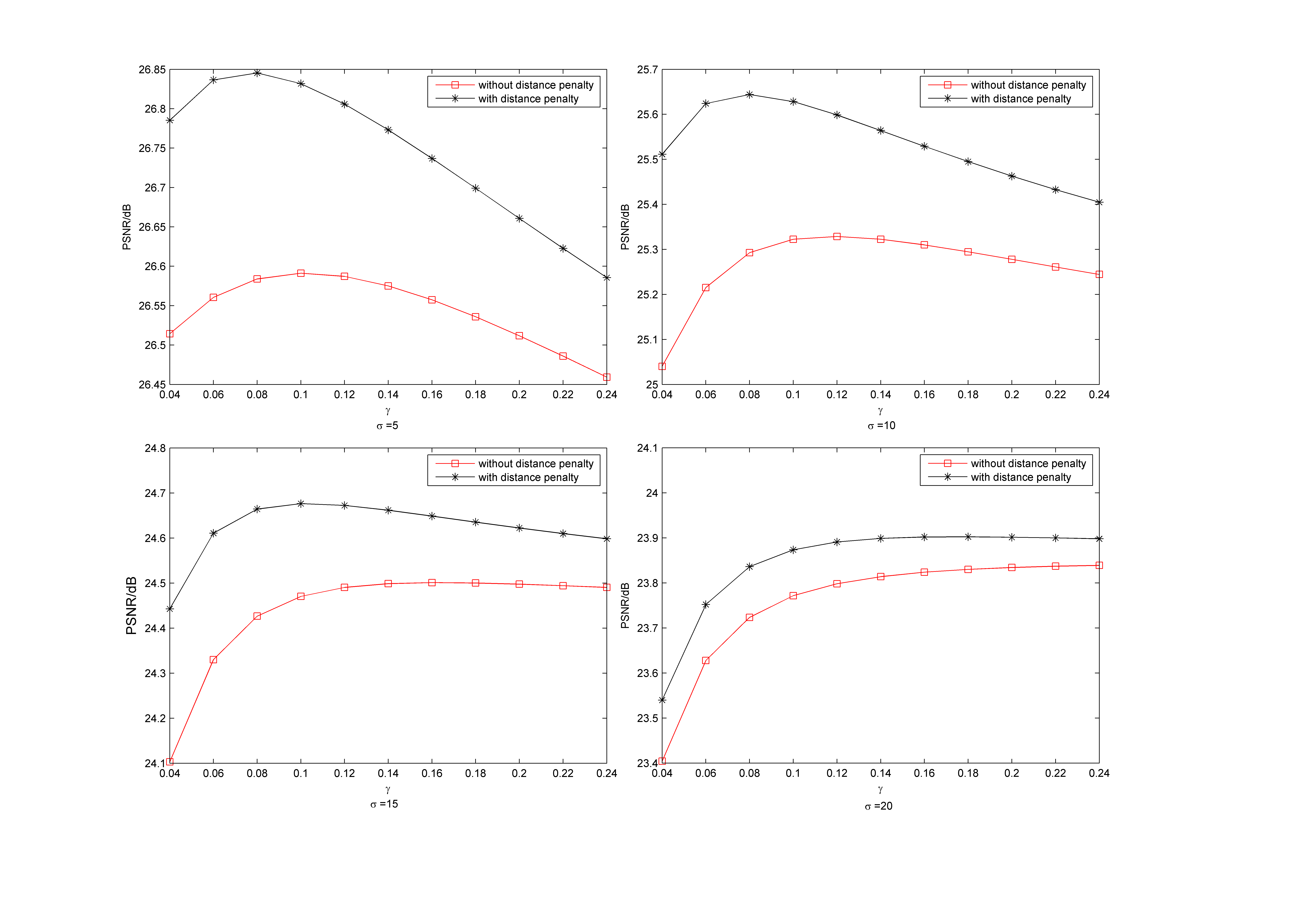

Supplement: S23 Fig — (TIF) [file pone.0182165.s023.tif]

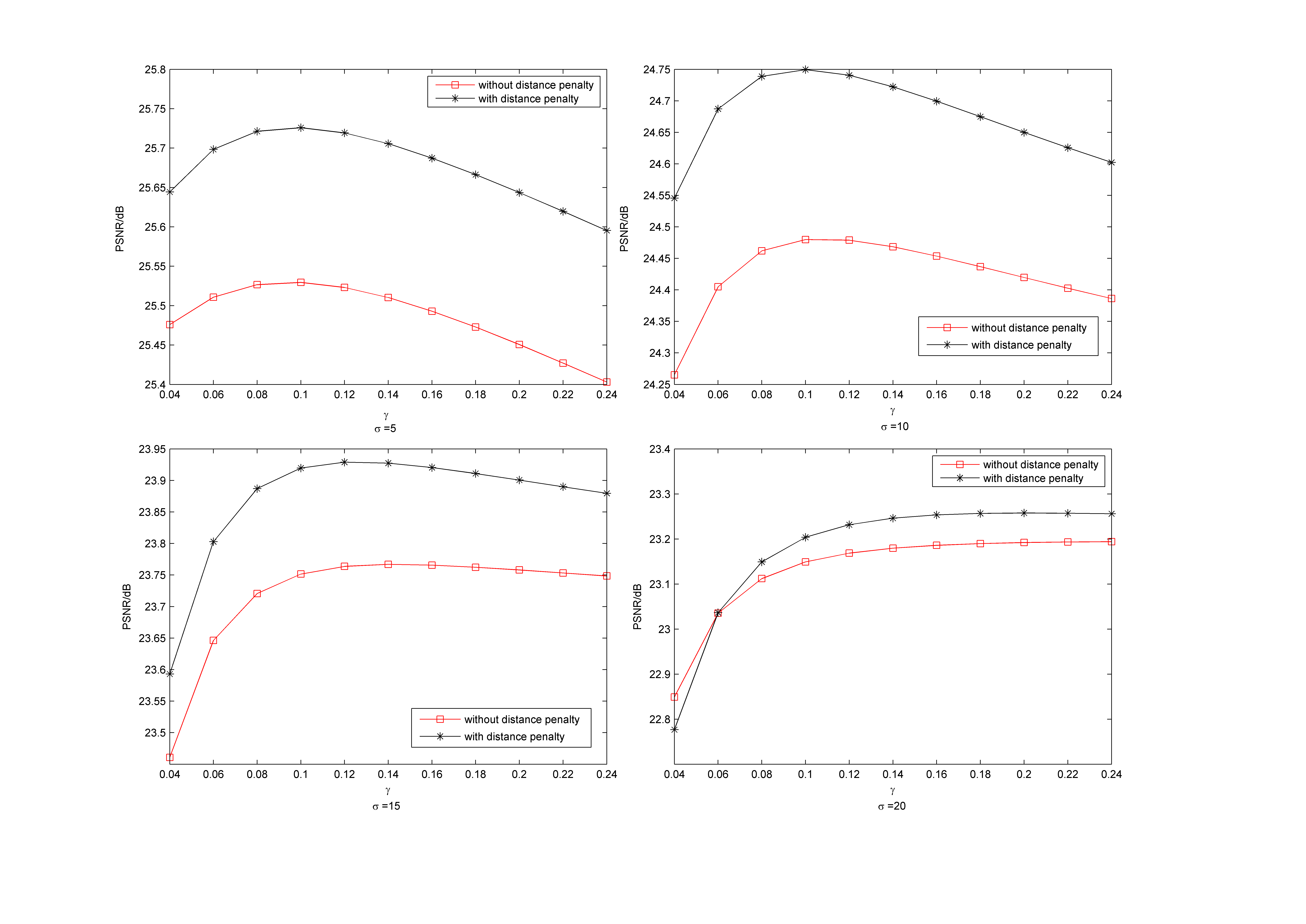

Supplement: S24 Fig — (TIF) [file pone.0182165.s024.tif]
