# Supplementary material for: Dictionary learning based noisy image super-resolution via distance penalty weight model
Source: PLoS One. 2017 Jul 31;12(7):e0182165. doi: 10.1371/journal.pone.0182165 (PMC5536359; doi:10.1371/journal.pone.0182165)
Supplement: S1 Table — (PDF) [file pone.0182165.s025.pdf]

S1 Table. Effect of IBP on Average PSNR (dB) and SSIM （Set14 and B100）

dataset	scale	IBP	$\sigma=5$		$\sigma=10$		$\sigma=15$		$\sigma=20$	
			PSNR	SSIM	PSNR	SSIM	PSNR	SSIM	PSNR	SSIM
Set14	×2	✗	29.29	0.793	26.58	0.643	24.33	0.521	22.48	0.427
		✓	28.33	0.732	24.51	0.527	21.63	0.391	19.39	0.301
		ours	**29.70**	**0.821**	**27.80**	**0.738**	**26.38**	**0.673**	**25.35**	**0.622**
	×3	✗	26.88	0.722	25.09	0.597	23.34	0.489	21.78	0.405
		✓	26.42	0.669	23.55	0.488	21.08	0.363	19.04	0.280
		ours	**27.17**	**0.740**	**25.78**	**0.666**	**24.67**	**0.608**	**23.77**	**0.558**
	×4	✗	25.49	0.666	24.07	0.563	22.59	0.469	21.21	0.393
		✓	25.20	0.622	22.84	0.464	20.63	0.349	18.74	0.270
		ours	**25.74**	**0.679**	**24.64**	**0.617**	**23.70**	**0.569**	**22.91**	**0.528**
B100	×2	✗	28.64	0.768	26.20	0.619	24.09	0.497	22.31	0.405
		✓	27.82	0.713	24.28	0.509	21.50	0.374	19.31	0.286
		ours	**28.95**	**0.792**	**27.29**	**0.704**	**26.08**	**0.638**	**25.20**	**0.587**
	×3	✗	26.56	0.685	24.88	0.562	23.21	0.456	21.68	0.374
		✓	26.12	0.636	23.39	0.458	20.98	0.337	18.98	0.256
		ours	**26.85**	**0.701**	**25.64**	**0.627**	**24.66**	**0.570**	**23.84**	**0.523**
	×4	✗	25.47	0.630	24.08	0.529	22.63	0.438	21.25	0.365
		✓	25.15	0.587	22.81	0.432	20.62	0.322	18.73	0.246
		ours	**25.72**	**0.642**	**24.74**	**0.582**	**23.89**	**0.536**	**23.15**	**0.498**
